# Recent Progress in Synthesis of Alkyl Fluorinated Compounds with Multiple Contiguous Stereogenic Centers

**DOI:** 10.3390/molecules29153677

**Published:** 2024-08-02

**Authors:** Xuemei Yin, Xihong Wang, Lei Song, Junxiong Zhang, Xiaoling Wang

**Affiliations:** 1 College of Pharmacy, Southwest Minzu University, Chengdu 610041, China; scsonglei@163.com (L.S.);; 2 Chengdu Institute of Biology, Chinese Academy of Sciences, Chengdu 610213, China; 3 University of Chinese Academy of Sciences, Beijing 100049, China

**Keywords:** alkyl fluorides, contiguous stereogenic centers, asymmetric synthesis, review

## Abstract

Organic fluorides are widely used in pharmaceuticals, agrochemicals, material sciences, and other fields due to the special physical and chemical properties of fluorine atoms. The synthesis of alkyl fluorinated compounds bearing multiple contiguous stereogenic centers is the most challenging research area in synthetic chemistry and has received extensive attention from chemists. This review summarized the important research progress in the field over the past decade, including asymmetric electrophilic fluorination and the asymmetric elaboration of fluorinated substrates (such as allylic alkylation reactions, hydrofunctionalization reactions, Mannich addition reactions, Michael addition reactions, aldol addition reactions, and miscellaneous reactions), with an emphasis on synthetic methodologies, substrate scopes, and reaction mechanisms.

## 1. Introduction

**Figure 1:**
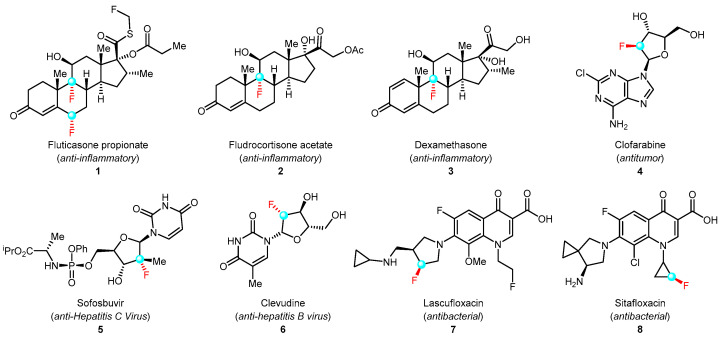
Representative drugs containing C(sp^3^)–F stereogenic centers.

The fluorine atom has the properties of strong electronegativity and a small atomic radius. The introduction of fluorine atoms into organic molecules can regulate the physicochemical and biological properties of the compounds, including the dipole moment, lipophilicity, metabolic stability, and bioavailability [1,2,3]. Notably, approximately 45% and 41% of small-molecule fluoro-pharmaceuticals were approved by the Food and Drug Administration (FDA) in 2018 and 2019, respectively [4,5,6]. Fluorinated compounds also play a crucial role in positron emission tomography (PET) [7,8,9,10] and materials science [11,12,13,14]. Especially, with the development of chiral fluorine chemistry and chiral drugs, fluorinated stereocenters have gradually become an important class of structural motifs widely used in medicinal chemistry and health sciences. For example, the glucocorticoid drugs fluticasone propionate (**1**, Figure 1), fludrocortisone acetate (**2**, Figure 1), and dexamethasone (**3**, Figure 1); the antichildhood leukaemia drug clofarabine (**4**, Figure 1); the antiviral drug sofosbuvir (**5**, Figure 1) against hepatitis C virus; the antiviral drug clevudine (**6**, Figure 1) against hepatitis B virus; and the antibacterial drugs lascufloxacin (**7**, Figure 1) and sitafloxacin (**8**, Figure 1) all contain C(sp^3^)–F stereocenters in their structures [3,5,6]. Therefore, it is of great significance to develop efficient synthetic methods for the preparation of chiral alkyl fluorides.

**Figure 2:**
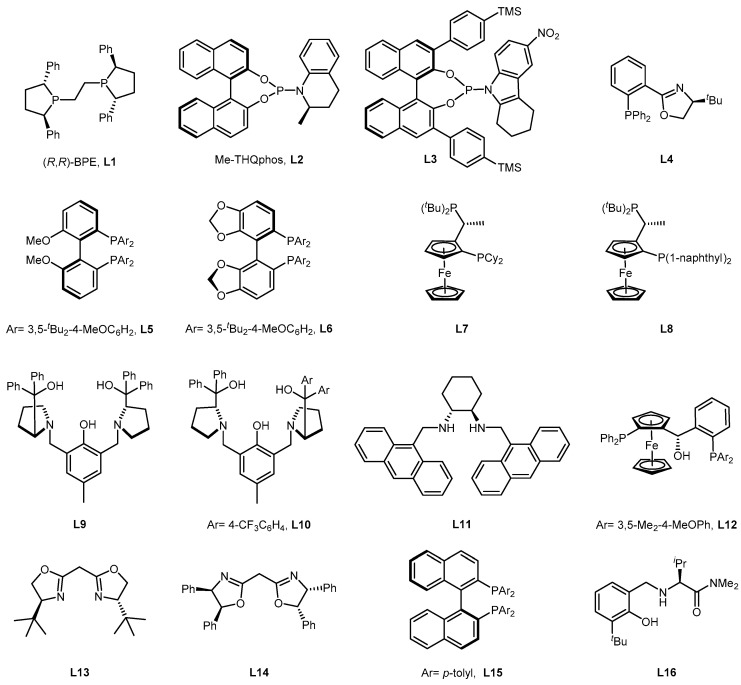
Chiral ligands for the asymmetric synthesis of alkyl fluorides bearing multiple contiguous stereogenic centers.

**Figure 3:**
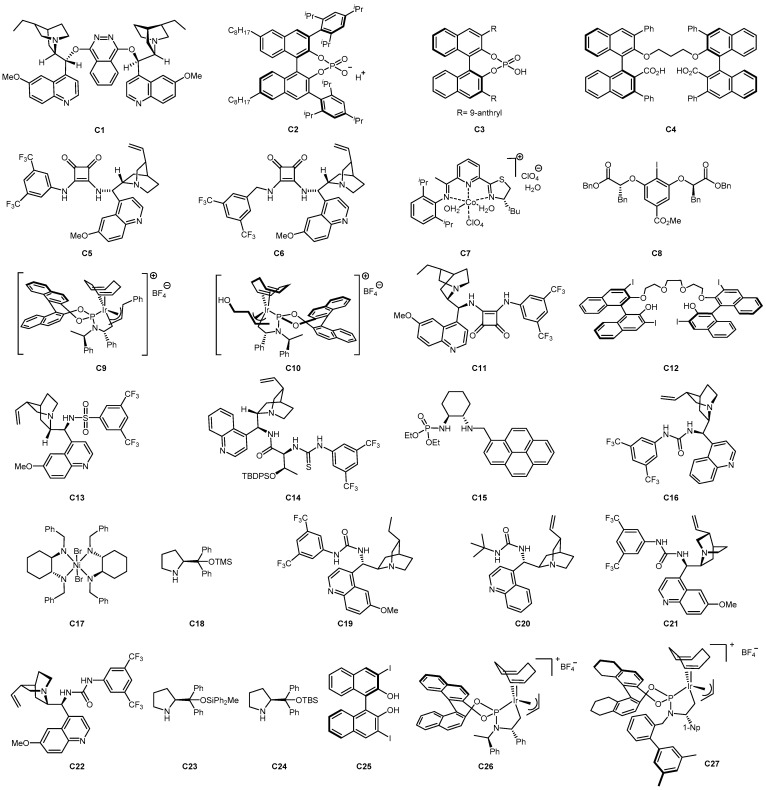
Chiral catalysts and metals complexes for the asymmetric synthesis of alkyl fluorides bearing multiple contiguous stereogenic centers.

In recent years, the flourishing development of fluorine chemistry has promoted remarkable advancements in the synthesis of chiral fluorinated compounds [15,16,17,18,19,20]. Innovative asymmetric catalytic methods for the synthesis of chiral alkyl fluorides continue to emerge, such as transition metal-catalyzed [21], organocatalyzed [22], high-valent iodine-catalyzed [23,24], and phase-transfer-catalyzed [25]. It is worth noting that the enantioselective construction of C(sp^3^)–F quaternary stereocenters is quite challenging. In 2018, Toste et al. provided a comprehensive review of modern methods for asymmetrically constructing C(sp^3^)–F quaternary stereocenters [26]. In 2019, Han et al. summarized cyclic fluorinated enolates generated in situ via detrifluoroacetylative for the preparation of compounds with quaternary C–F stereogenic carbon [27]. In 2020, Granado and Vallribera published a review on the preparation of *α*-quaternary fluorinated *β*-keto esters via asymmetric fluorination of *β*-keto esters [28]. In 2021, Hartwig et al. reported an elegant review on transition metal-catalyzed monofluoroalkylation: the synthesis of alkyl fluorides via C–C bond formation [29]. However, the synthesis of chiral alkyl fluorides bearing multiple contiguous stereogenic centers in a highly enantio- and diastereoselective manner remains a remarkably challenging task, and methods supporting this goal are rare, mainly due to the inherent difficulties of constructing the C(sp^3^)–F bond and controlling enantio- and diastereoselectivities simultaneously. Notably, intense efforts toward this goal have been seen in recent years. It was found that a variety of chiral ligands (**L1**–**L16**, Figure 2), including bisphosphine ligands (**L1**, **L5**, **L6**, **L15**); phosphoramidite ligands (**L2**, **L3**); phosphine-oxazoline ligand (**L4**); ferrocenyl ligands (**L7**, **L8**, **L12**); prophenol ligands (**L9**, **L10**), diamine ligand (**L11**); bisoxazoline ligand (**L13**, **L14**); aminophenol (**L16**); and metal complexes and chiral catalysts (**C1**–**C27**, Figure 3), for example, cinchona alkaloid catalyst (**C1**), anion phase-transfer catalysts (**C2**–**C4**), quinine squaramide catalysts (**C5**, **C6**, **C11**), aryl iodide organocatalyst (**C8**), quinine-derived sulfonamide (**C13**), amine phosphoramide (**C15**), etc., could effectively promote the asymmetric synthesis of those compounds. Herein, the recent advances in the highly enantio- and diastereoselective synthesis of alkyl fluorides bearing multiple consecutive stereocenters are summarized, including asymmetric electrophilic fluorination and asymmetric elaboration of fluorinated substrates (such as allylic alkylation reactions, hydrofunctionalization reactions, Mannich addition reactions, Michael addition reactions, aldol addition reactions, and miscellaneous reactions). The synthetic methodologies, substrate scopes, and reaction mechanisms are mainly discussed.

## 2. Asymmetric Electrophilic Fluorination

**Figure 4:**
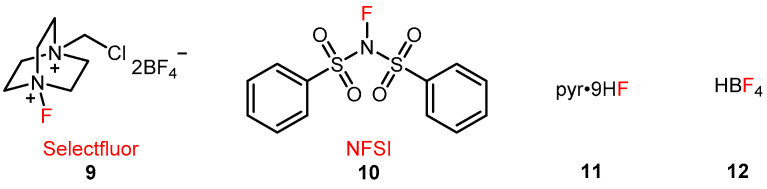
The common electrophilic and nucleophilic fluorinating reagents used for catalytic asymmetric fluorination.

The development of readily available and efficient sources of electrophilic fluorinating reagents, such as 1-chloromethyl-4-fluoro-1,4-diazoniabicyclo[2.2.2]octane bis(tetrafluoroborate) (Selectfluor) (**9**, Figure 4) [30] and *N*-fluorobenzenesulfonimide (NFSI) (**10**, Figure 4) [31], has had a significant impact on catalytic asymmetric electrophilic fluorination, and encouraging progress has been made. In contrast, the synthesis of chiral alkyl fluorides with multiple contiguous stereogenic centers using nucleophilic fluorinating reagents is more difficult due to the poor reactivity of the fluorine anion. Asymmetric electrophilic fluorination catalyzed by transition metal catalysts or chiral organocatalysts is one of the typical methods used for the synthesis of chiral alkyl fluorinated compounds with multiple contiguous stereogenic centers containing a C–F quaternary carbon. The diastereo- and enantioselective electrophilic fluorination mainly includes asymmetric fluorinative dearomatization, sequential asymmetric addition/fluorination, and asymmetric cyclization/fluorination. 

**Scheme 1:**
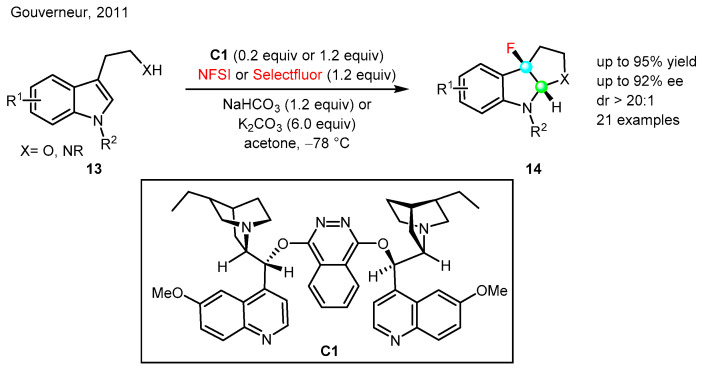
Asymmetric dearomatization of indole derivatives via cascade fluorocyclization [40].

**Scheme 2:**
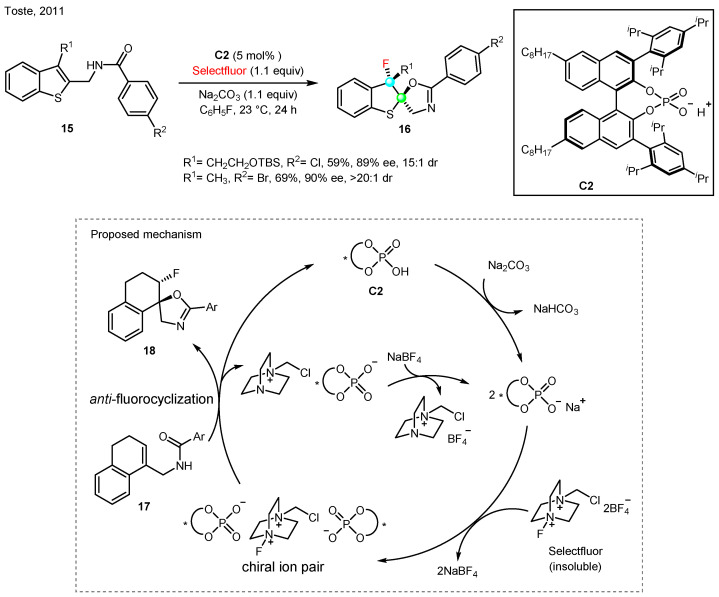
Phase transfer-catalyzed asymmetric dearomatized fluorocyclizations of benzothiophene derivatives [25].

**Scheme 3:**
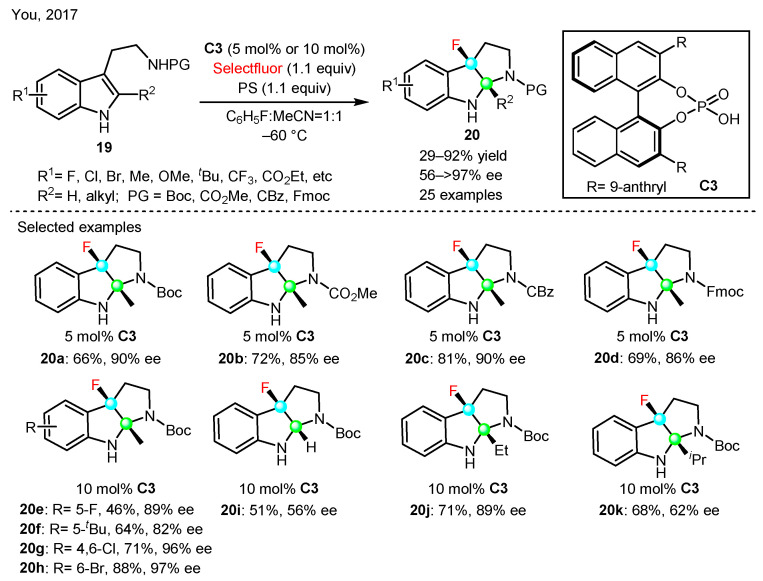
Asymmetric fluorinative dearomatization of tryptamine derivatives [41].

**Scheme 4:**
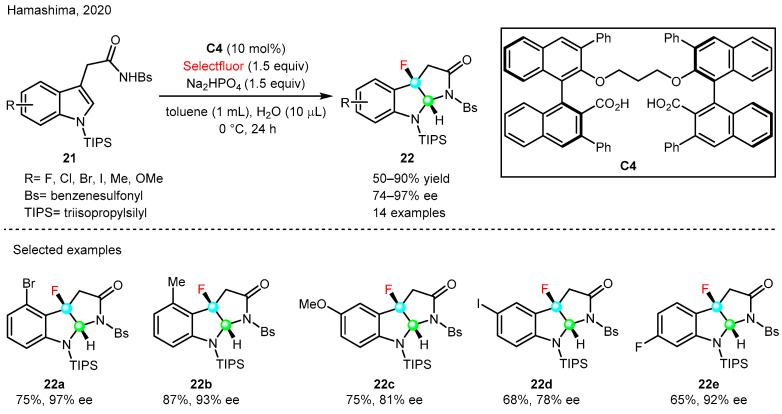
Phase transfer-catalyzed asymmetric dearomatized fluoroamidation of indole derivatives [42].

Catalytic asymmetric dearomatization (CADA) [32,33,34,35,36,37,38,39] reactions via fluorination represent a powerful strategy for constructing chiral fluorine-containing compounds from readily available aromatic derivatives. In 2011, Gouverneur et al. reported the first organocatalyzed asymmetric dearomatization of indole derivatives **13** via cascade fluorocyclization utilizing a cinchona alkaloid **C1** as a catalyst and NFSI or Selectfluor as an electrophilic reagent (Scheme 1) [40]. Enantioenriched fluorinated heterocycles **14** were obtained with moderate to high enantioselectivities. Almost at the same time, Toste et al. developed asymmetric electrophilic fluorination using a chiral anion phase-transfer catalyst **C2**. Interestingly, benzothiophenes substrates **15** were converted to desired fluorocyclization products **16** in high optical purity and good yield through CADA reactions via fluorination (Scheme 2) [25]. The proposed catalytic mechanism is shown in Scheme 2. Phosphate (2 equiv.) undergoes salt substitution with Selectfluor to produce the chiral ion pair, which is soluble in nonpolar solvents, and then the chiral ion pair can mediate the fluorocyclization of the substrate **17**. In 2017, You et al. developed asymmetric fluorinative dearomatization of tryptamine derivatives **19** under anion phase-transfer catalysis, which entailed the use of Selectfluor as an electrophilic fluoride source and a chiral phosphate anion derived from BINOL backbone **C3** as a catalyst (Scheme 3) [41]. They noticed that a proton sponge (PS) can effectively improve the reaction yield. A variety of fluorinated pyrroloindolines **20** bearing two contiguous quaternary stereogenic centers were obtained with excellent enantioselectivities (up to 97% ee) and high yields (up to 92% yield). Regarding the exploration of the substrates’ scope, tryptamines with electron-withdrawing protecting groups (Boc, CO_2_Me, Cbz, and Fmoc) were well tolerated. *N*-Boc protected tryptamines with different electron-withdrawing (5-CO_2_Et, 5-CF_3_, 5-Cl, 5-Br, and 5-F) and electron-donating (5-MeO, 5-CH_3_, and 5-*^t^*Bu) substituents at the C5 position, which proceeded with excellent enantioselectivity. 4,6-dihalo-substituted substrates and 2- or 6-substituent of the indole moiety were also well tolerated to provide target products with high enantioselectivity. Substrates bearing higher steric hindrance substituents or simple H and methyl at the C7 position resulted in moderate enantioselectivity. Control experiments suggested that the reaction proceeded via bifunctional activation using a chiral BINOL-derived phosphate anion **C3**. Recently, the asymmetric dearomatizing fluoroamidation of indole acetamide derivatives **21** using a dicarboxylate phase-transfer catalyst **C4** under mild conditions was developed by Hamashima et al. (Scheme 4) [42]. Indoles with various substitution patterns were suitable substrates, providing easy access to chiral fluoropyrroloindoline derivatives **22** in 50–90% yields and 74–97% ee. It is worth mentioning that the addition of an appropriate amount of water to the reaction system was essential to facilitate the reaction and ensure reproducibility.

**Scheme 5:**
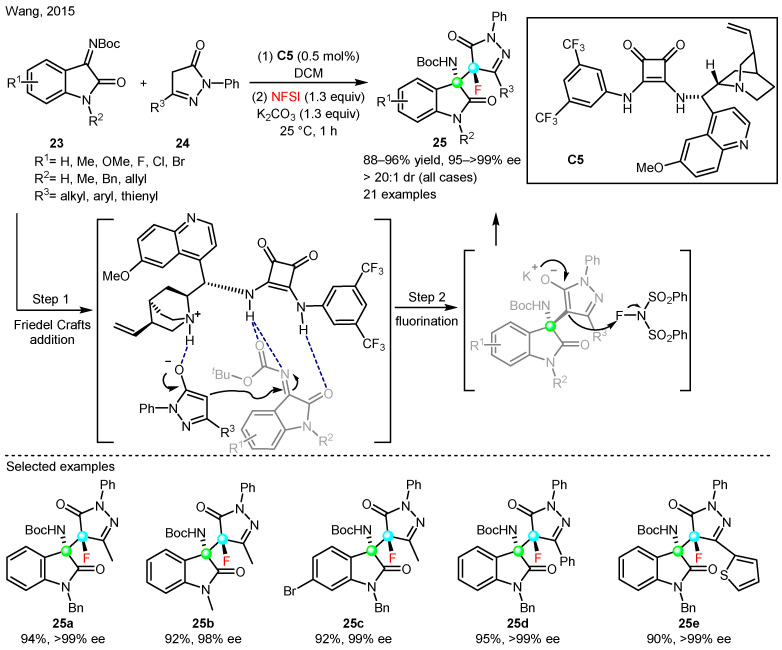
Organocatalytic asymmetric Friedel–Crafts addition/fluorination [43].

**Scheme 6:**
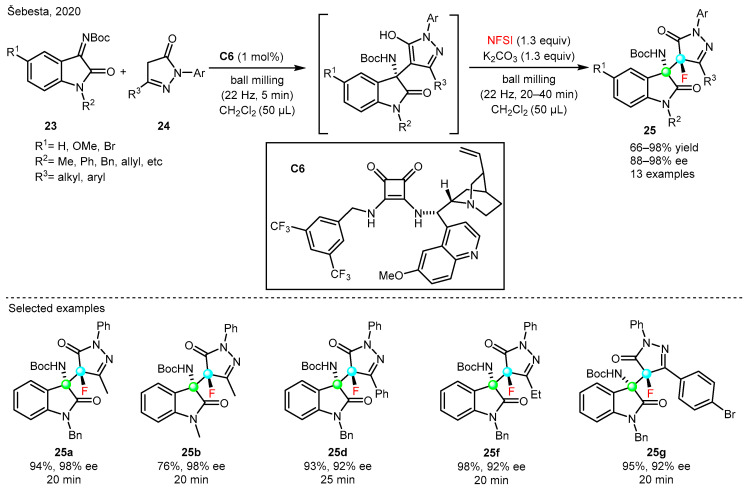
Mechanochemically activated asymmetric organocatalytic domino Mannich reaction/fluorination [44].

In 2015, Wang et al. reported a one-pot reaction sequence of the organocatalytic asymmetric Friedel–Crafts addition of 4-nonsubstituted pyrazolones **24** to isatin-derived *N*-Boc ketimine **23** followed by diastereoselective electrophilic fluorination (Scheme 5) [43]. By using 0.5 mol% quinine squaramide **C5** as a catalyst, CH_2_Cl_2_ as a solvent in the first step, NFSI as an electrophilic reagent, and K_2_CO_3_ as a base in the second step, a variety of fluorinated oxindole–pyrazolone adducts **25** bearing vicinal tetrasubstituted stereocenters containing a C–F quaternary carbon were obtained with 88–96% yields, 95–99% ee, and >20:1 dr. In 2020, Šebesta and co-workers reported the squaramide **C6**-catalyzed asymmetric Mannich reaction of oxindole imines **23** with pyrazolones **24**, followed by electrophilic diastereoselective fluorination (Scheme 6) [44]. The reaction proceeded under liquid-assisted grinding conditions to provide oxindolyl fluoropyrazolone derivatives **25** with enantiomer purities up to a 99:1 enantiomeric ratio. The organocatalytic Domino Mannich reaction was also suitable for isoxazole, but the yield was slightly lower, with up to 52% ee. However, thiazolones and oxazolones were ineffective substrates. Notably, the ball-milling reaction was more efficient than that of the solution reaction. The ball milling reaction time could be greatly shortened to a few minutes, and the amount of solvent required for the reaction could be minimized.

**Scheme 7:**
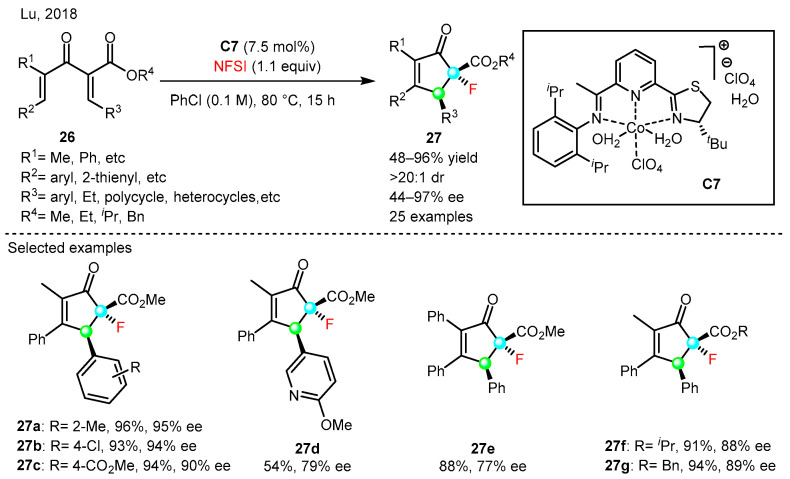
Cobalt-catalyzed enantioselective sequential Nazarov cyclization/electrophilic fluorination [45].

In 2018, Lu et al. reported an enantioselective sequential Nazarov cyclization/electrophilic fluorination of divinyl ketone derivatives **26** catalyzed by the thiazoline iminopyridine (TIP)-Co complex **C7**, yielding various substituted chiral α-fluorinated cyclopentenones **27** with 48–96% yields, 44–97% ee, and >20:1 dr (Scheme 7) [45]. Further derivatization of the α-fluorinated cyclopentenones allowed the construction of chiral cyclopentenols with three contiguous stereocenters and the synthesis of chiral α-single fluorine substituted cyclopentenones. Additionally, the gram-scale experiment proceeded well with little loss of ee values for the target compounds. 

**Scheme 8:**
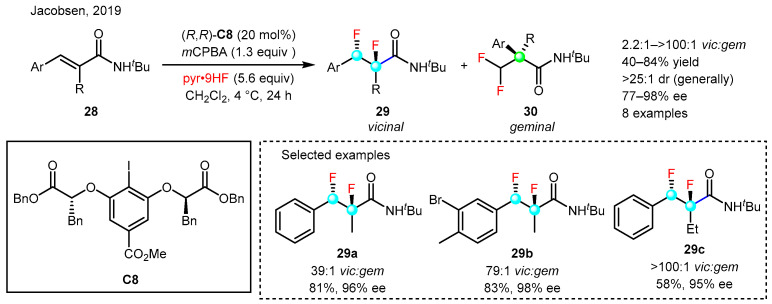
Enantioselective 1,2-difluorination of cinnamamides catalyzed by chiral aryl iodide [46].

**Scheme 9:**
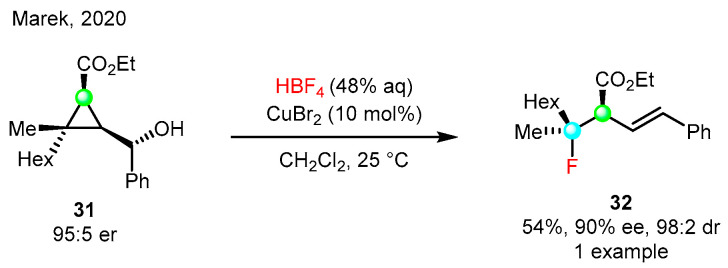
Enantio- and diastereoselective synthesis of acyclic compound possessing tertiary alkyl fluoride and ester [47].

We also note two particular examples of the synthesis of chiral alkyl fluorides with two adjacent chiral centers by using nucleophilic fluorinating reagents. In 2019, Jacobsen et al. reported a diastereo- and enantioselective 1,2-difluorination of cinnamamides utilizing pyr·9HF (**11**) as a fluoride source mediated by chiral aryl iodide organocatalyst **C8** and *m*-CPBA as a stoichiometric oxidant (Scheme 8) [46]. This method afforded 1,2-difluoride-containing compounds with 1,2-/1,1-difluorination selectivity of 2.2:1–>100:1, in yields of 40–84%, ee of 77–98%, and dr generally > 20:1. Selectivity for 1,2-difluorination versus 1,1-difluorination resulting from phenonium rearrangement was enforced through anchimeric assistance by a proximal *N*-*tert*-butyl amide substituent. In 2020, Marek and co-workers reported that optically pure cyclopropyl carbinol derivatives **31** undergo a stereo- and regioselective nucleophilic substitution at the quaternary carbon center with a pure conformational inversion, yielding tertiary alkyl fluoride and ester derivatives **32** as a single diastereomer (Scheme 9) [47].

## 3. Asymmetric Elaboration of Fluorine-Containing Substrates

Asymmetric allylic alkylation reactions, Mannich addition reactions, Michael addition reactions, aldol reactions, and cross-coupling reactions of fluorine-containing substrates represent another classical approach for constructing multiple contiguous stereocenters containing C-F stereocenters.

### 3.1. Allylic Alkylation Reactions

Transition metal-catalyzed asymmetric allylic substitution reactions [48,49,50,51,52,53,54,55,56,57,58,59] are one of the most powerful methods for building up stereocenters. Asymmetric allylic alkylation reactions of prochiral fluorine-containing nucleophiles provide an efficient and reliable strategy for the synthesis of chiral alkyl fluorinated molecules bearing two or more vicinal stereogenic centers.

**Scheme 10:**
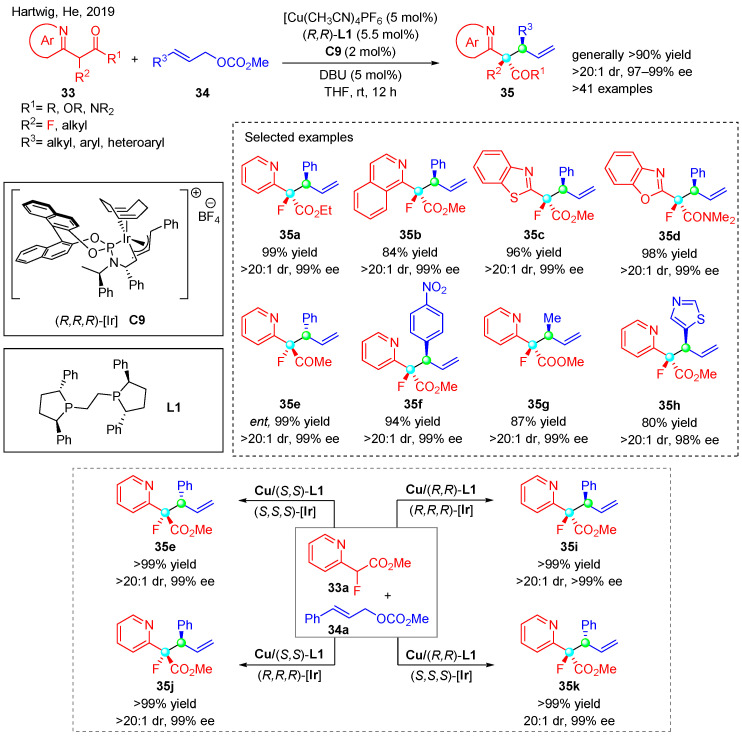
Copper- and iridium-catalyzed asymmetric allylic alkylation of α-fluorinated azaaryl acetates with cinnamyl methyl carbonate [60].

*α*-Pyridine-*α*-fluoro esters are important synthons for the synthesis of chiral molecules containing both a fluorine atom and a pyridine ring. However, *α*-pyridine-*α*-fluoro esters are less nucleophilic, and the potential inactivation or poisoning of catalysts through the coordination of heteroaromatics will result in poor control of reactivity and stereoselectivities. In 2019, Hartwig, He and co-workers reported the stereodivergent allylic alkylation of *α*-fluorinated azaaryl acetates **33** with cinnamyl methyl carbonates **34** utilizing the combination of a chiral cyclometalated iridium catalyst **C9** and a Cu complex ligated to (*R*,*R*)-BPE (**L1**) (Scheme 10) [60]. In the presence of 1,8-diazabicyclo [5.4.0]undec-7-ene (DBU) as a base, the allylic substitution reaction gave fluorinated products within vicinal quaternary and tertiary stereogenic centers in yields generally >90%, >20:1 dr, and 97–99% ee. A variety of alkyl- and aryl-substituted allylic esters, *α*-fluorinated azaaryl acetates, ketones, and amides were amenable to this Cu/Ir catalytic system, giving the desired products with satisfactory results. Notably, the copper catalyst and iridium catalyst could, respectively, control the configuration of the nucleophilic carbon of an unstabilized enolate and the electrophilic carbon of allylic carbonates in this reaction system.

**Scheme 11:**
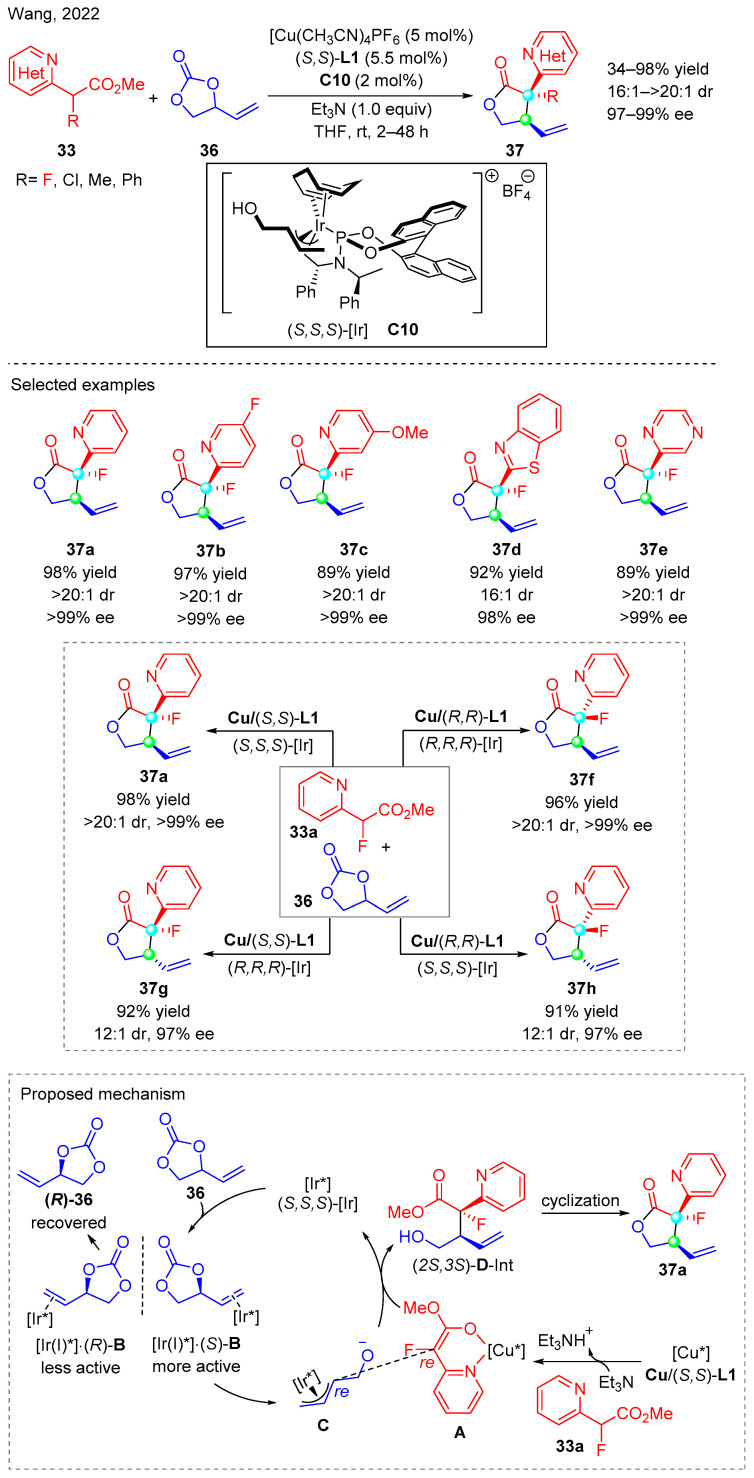
Copper- and iridium-catalyzed cascade allylic alkylation/lactonization of *α*-fluoro-*α*-azaaryl acetates with vinylethylene carbonates [61].

In 2022, Wang and co-workers described a dual Cu/Ir catalytic system for the asymmetric [3+2] annulation of *α*-fluoro-*α*-azaaryl acetates **33** with vinylethylene carbonates **36** via a cascade allylic alkylation/lactonization reaction (Scheme 11) [61]. In the presence of Cu(CH_3_CN)_4_PF_6_ and **C10** as a catalyst, (*S*,*S*)-**L1** as ligand, Et_3_N as a base, *α*-fluoro-*γ*-butyrolactones **37** containing vicinal stereogenic centers were obtained with 34–98% yields, 16:1–>20:1 dr, and 97–99% ee. It is worth noting that all four possible stereoisomers of these valuable products could be readily accessed individually via simple permutations of two chiral catalysts. The proposed mechanism for this [3+2] annulation reaction is shown in Scheme 11. Under basic conditions, chiral Cu(I)-(*S*,*S*)-**L1** could coordinates with the nitrogen atom of the azaarene ring and carbonyl oxygen in *α*-fluoro-*α*-azaryl acetate to generate the chiral metalated intermediate **A**. Simultaneously, the coordination of [(*S*,*S*,*S*)-Ir(I)] **C10** with racemic vinylethylene carbonate(±) generated the corresponding complexes [Ir(I)∗]·(*R*)-**B** and [Ir(I)∗]·(*S*)-**B**. (*R*)-vinylethylene carbonate (*R*)-**36** could be recovered from the less reactive former species through kinetic resolution, while the latter more reactive species was prioritized to undergo decarboxylative oxidation addition and generate the zwitterionic Ir(III)-*π*-allyl species **C** with the configuration inversion. The chiral metalated intermediate **A** then nucleophilically attacked the Ir(III)-*π*-allyl species **C** in a highly *Re*-*Re* stereoselective manner to form the allylic alkylation intermediate (*2S*,*3S*)-**D**-Int and regenerate the chiral Cu/Ir catalysts. Finally, the intermediate (*2S*,*3S*)-**D**-Int underwent intramolecular esterification to access target products.

**Scheme 12:**
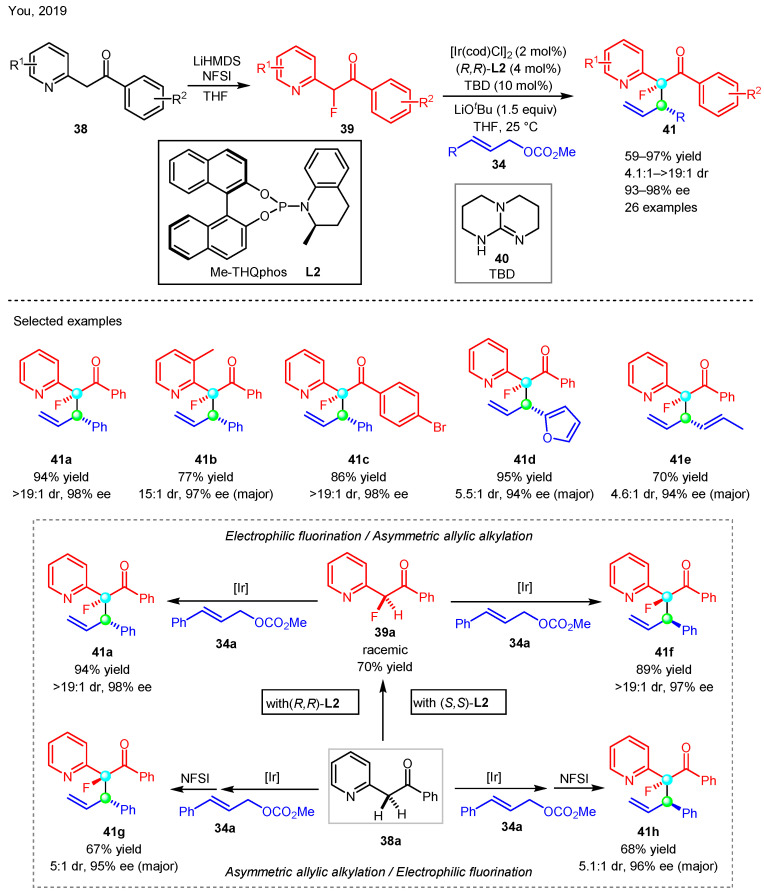
Iridium-catalyzed asymmetric allylic alkylation/fluorination of acyclic ketones [62].

In 2019, You et al. reported iridium-catalyzed asymmetric allylic alkylation/fluorination of acyclic ketones **38** (Scheme 12) [62]. *α*-pyridyl-*α*-fluoroketones **41** bearing vicinal tertiary and fluorine-containing quaternary stereocenters were obtained in 59–97% yields, 4.1:1–>19:1 dr, and 93–98% ee by utilizing Me-THQphos (*R*,*R*)-**L2** as a chiral ligand in the presence of *^t^*BuOLi and 1,5,7-triazabicyclo [4.4.0] dec-5-ene (TBD) in THF at room temperature. Distinct from the above-mentioned stereodivergent synthesis strategies reported by Hartwig [60] and Wang [61], which generally required the use of two different chiral catalysts (Cu/Ir), only one single chiral iridium catalyst was required in You’s work, providing an elegant pathway for the stereodivergent synthesis of molecules bearing multiple consecutive stereocenters. All four possible stereoisomers of the desired products were prepared from the same pyridone derivatives as substrates by simply adjusting the sequence of fluorination and allylic alkylation and changing the absolute configuration of the iridium catalyst.

**Scheme 13:**
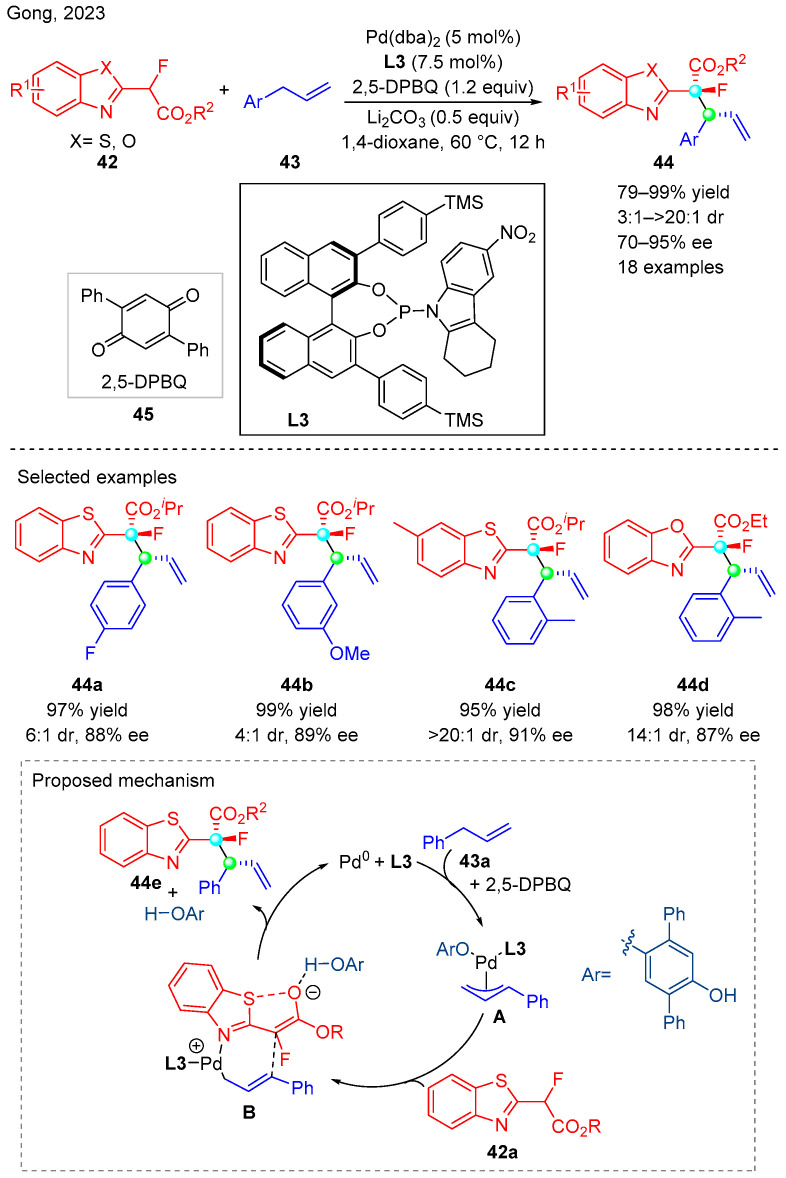
Palladium-catalyzed asymmetric allylic C–H alkylation reaction of allylarenes and *α*-fluorinated *α*-benzothiazylacetates [63].

In 2023, Gong and co-workers developed a branch- and asymmetric allylic C–H alkylation reaction of allylarenes **43** and *α*-fluorinated *α*-benzothiazylacetates **42** catalyzed by chiral phosphoramidite (**L3**)-palladium catalysis in the presence of Li_2_CO_3_ and 2,5-diphenylquinone (2,5-DPBQ) in 1,4-dioxane at 60 °C (Scheme 13) [63]. Under the optimized reaction conditions, varieties of *α*-quaternary fluorinated ester products **44** were obtained in yields of 79–99%, dr of 3:1–20:1, and ee of 70–95%. A catalytic cycle was proposed in Scheme 13. In the presence of a Pd-**L3** catalyst and 2,5-DPBQ, allylbenzene underwent allylic C–H bond cleavage via a concerted proton and two-electron-transfer process to form the *π*-allylpalladium intermediate **A**. Then, deprotonation of *α*-fluorinated *α*-benzothiazylacetate and *π*-*σ* isomerization of the allyl skeleton generated the ion pair complex **B**, wherein 2,5-DPBQ might be adjacent to the enolate anion via hydrogen-bonding interaction. Finally, the intermediate **B** underwent an inner-sphere allylation to yield the desired product and regenerate the Pd-**L3** catalyst.

**Scheme 14:**
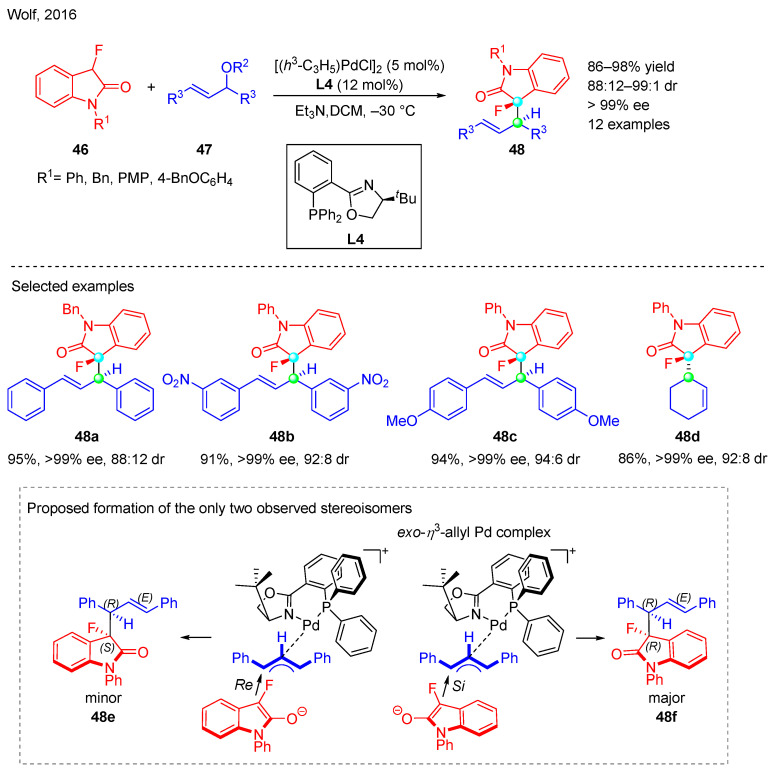
Palladium-catalyzed asymmetric fluoroenolate alkylation with allyl acetate/carbonate [64].

We also noted that Wolf et al. reported asymmetric fluoroenolate alkylation with allyl acetate/carbonate **47** under palladium catalysis using (*S*)-*t*-Bu-PHOX (**L4**) as a chiral ligand (Scheme 14) [64]. Versatile 3, 3-disubstituted fluorooxindoles **48** bearing vicinal chirality centers were synthesized in 86–98% yields, 88:12–>99:1 dr, and ≥99% ee. In addition, the regioselective asymmetric alkylation of nonsymmetrically substituted allylic acetates was studied, and the corresponding products were obtained with excellent regioselectivities. The stereochemical outcome of this reaction can be rationalized by a preferential attack occurring from the *Si* face of the fluoroenolate at the allylic position that is *trans* to the P atom of the **L4** in a favored *exo*-*η*^3^-allyl Pd complex. This pathway provides the (*R*,*R*,*E*) isomer **48f** as the major product, whereas an attack originating from the *Re* face of the fluoroenolate results in the formation of the minor (*S,R,E*) diastereomer **48e** (Scheme 14).

**Scheme 15:**
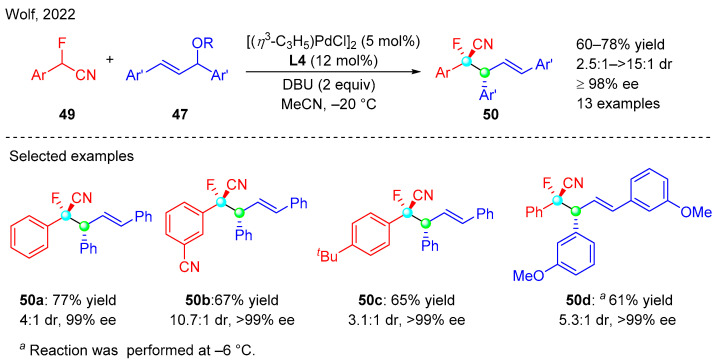
Palladium-catalyzed asymmetric allylic alkylation with *α*-aryl-*α*-fluoroacetonitriles [70].

The preparation of arylfluoroacetonitrile derivatives bearing two or more vicinal stereogenic centers via enantioselective C–C bond formation using arylfluoroacetonitrile as a starting material is challenging due to the relatively low C–H acidity of arylfluoroacetonitriles, the possibility of decomposition or HF elimination the side reaction of fluoroacetonitrile under basic conditions, and the difficulty in controlling the stereoselectivity of *α*-fluoronitrile carbanions [65,66,67,68,69]. To tackle the aforementioned challenges, Wolf and co-workers developed a palladium-catalyzed asymmetric allylic alkylation with *α*-aryl-*α*-fluoroacetonitriles **49** in the presence of phosphinoxazoline ligand **L4** and DBU at −20 °C in acetonitrile (Scheme 15) [70]. This reaction afforded the target product **50** featuring two contiguous chirality centers in 60–78% yields, 5:1–>15:1 dr, and ≥98% ee. The gram-scale synthesis of this method was also carried out, and good yield and stereochemical outcomes (>99% ee) were obtained. Additionally, asymmetric allylic alkylation products can be further derivatized via Stille cross-coupling reaction without obvious HF elimination.

### 3.2. Hydrofunctionalization Reactions

**Scheme 16:**
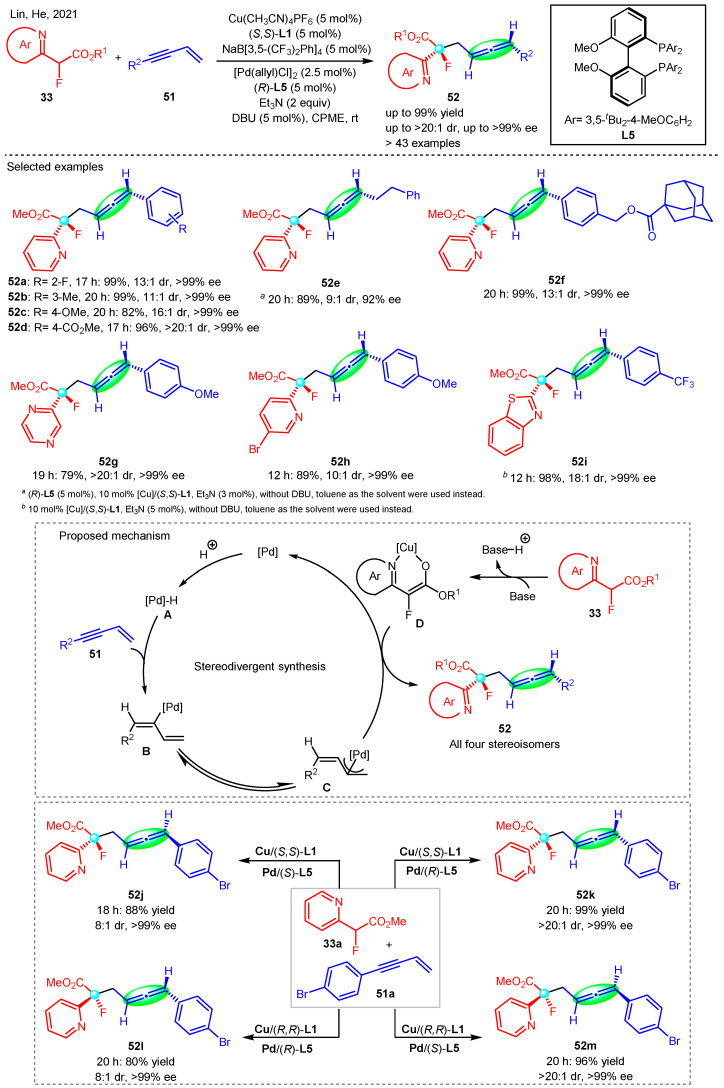
Copper- and palladium-catalyzed asymmetric hydrofunctionalization of conjugated enyenes [86].

Transition metal-catalyzed enantioselective hydrofunctionalization of alkenes, 1,3-dienes, allenes, and conjugated alkynes were discovered as efficient routes for the construction of a stereogenic center or axis [71,72,73,74,75,76,77,78,79,80,81,82,83,84,85]. In 2021, Lin, He and co-workers reported a novel Cu/Pd synergistic catalysis strategy for diastereoselective hydrocarbonation of conjugated alkynes (Scheme 16) [86]. The coupling of α-fluorinated azaaryl esters **33** with conjugated enynes **51**, in the presence of Cu(CH_3_CN)_4_PF_6_, (*S*,*S*)-BPE [(*S*,*S*)-**L1**], [Pd(allyl)Cl]_2_, **L5**, NaBArF_4_, Et_3_N, and DBU, provided tertiary fluoride-tethered allenes **52** containing a stereogenic center and axis with up to 99% yield, >20:1 dr, and >99% ee. This reaction has been shown to have a broad substrate scope. A variety of electron-withdrawing and electron-donating groups at different positions on the aryl units in conjugated enynes; a series of *α*-fluoro nucleophiles bearing quinolinyl, pyrazinyl, pyridinyl, benzothiazolyl and pyrimidinyl groups; and diverse natural and biologically active compounds were well tolerated. The proposed mechanism cycle is shown in Scheme 16. The enyne would initially insert regioselectively into the Pd-H generated in situ, yielding an allylpalladium species. Concurrently, the Cu-coordinated fluorinated enolate would act as an outer-sphere nucleophile. All four possible stereoisomers of the tertiary fluoride-tethered allenes were precisely synthesized with good diastereoselectivities and excellent enantioselectivities by simply permutating the absolute configuration of the ligand of Cu/Pd catalysts.

**Scheme 17:**
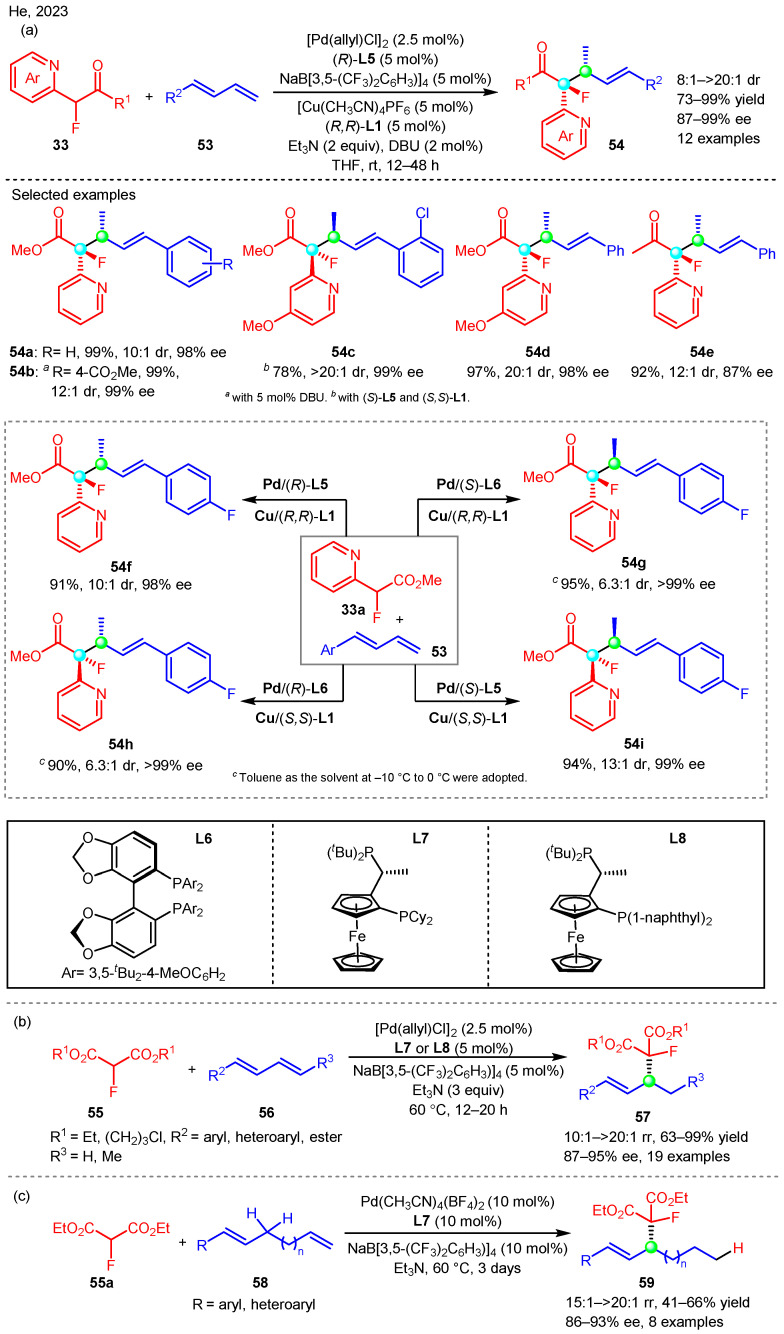
Asymmetric hydromonofluoroalkylation of 1,3-diene (**a**), monosubstituted and internal dienes (**b**) and skipping dienes (**c**) [87].

Recently, the same group employed a similar strategy to realize an asymmetric hydromonofluoroalkylation reaction of 1,3-diene **53** (Scheme 17a) [87]. A series of alkyl fluorine derivatives **54**, bearing a quaternary F-stereogenic center and vicinal tertiary stereogenic carbon center, were synthesized in yields up to 99%, >20:1 dr, and >99% ee. Intriguingly, all four stereoisomers of corresponding products can also be stereodivergently synthesized through Cu/Pd co-catalysis by using **L1**, **L5**, or **L6** as chiral ligands. The Pd/**L7** or Pd/**L8-**catalyzed asymmetric hydromonofluoroalkylation reaction of monosubstituted and internal dienes **56** was also developed to access alkyl fluorides in up to 99% yield, >20:1 rr, and 95% ee (Scheme 17b). In addition, asymmetric migratory monofluoroalkylation of skipping dienes **58** was established to realize the straightforward allylic C–H fluoroalkylation (Scheme 17c). Pleasantly, a variety of enantioenriched fluorinated rings can be obtained by diverse transformations.

### 3.3. Mannich Addition Reactions

**Scheme 18:**
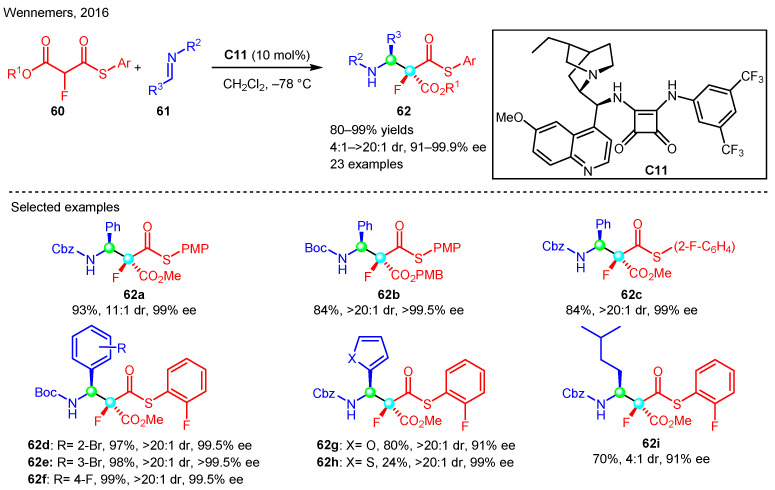
Asymmetric organocatalyzed addition reactions of *α*-fluorinated monothiomalonates to protected imines [93].

**Scheme 19:**
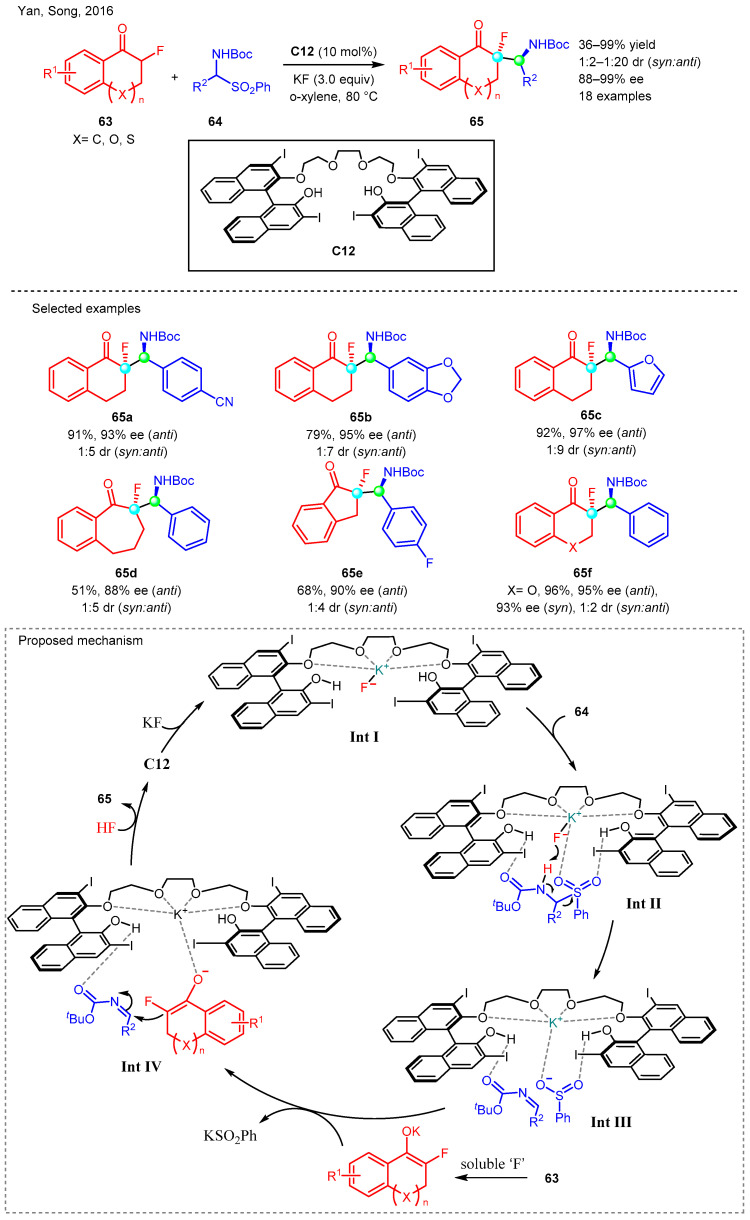
Organocatalyzed Mannich reaction of *α*-fluoro cyclic ketones with *α*-amidosulfones [94].

**Scheme 20:**
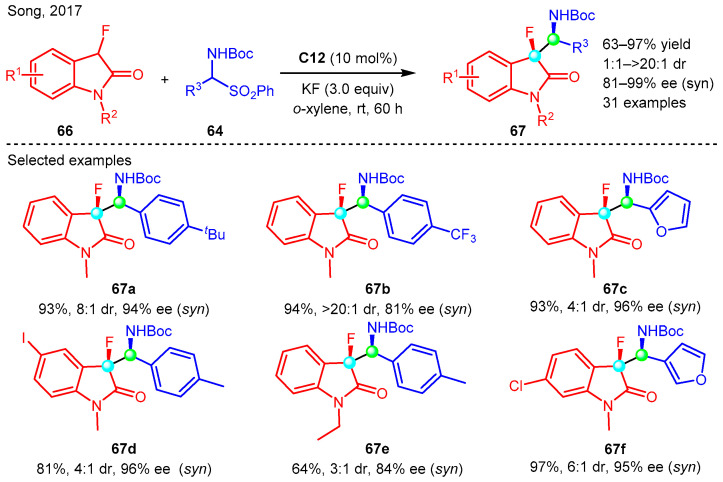
Asymmetric Mannich reactions of 3-fluoro-oxindoles with *α*-aminosulfones [95].

The catalytic asymmetric Mannich reaction of fluorinated nucleophiles provides an efficient route for the synthesis of pharmaceutically important fluorinated amino compounds [88,89]. The efficient organocatalytic asymmetric Mannich reaction of *α*-fluorinated *β*-ketoesters, *α*-fluorinated *β*-keto acyloxazolidinone, and *α*-fluorinated aromatic ketones for the synthesis of corresponding products featuring fluorinated tetrasubstituted carbon centers have been reported by Huang, Lu [90] and Jiang, Tan et al. [91,92]. In 2016, Wennemers and co-workers reported asymmetric addition reactions of α-fluorinated monothiomalonates **60** to protected imines **61**, providing various acyclic α-fluorinated *β*-amino thioesters **62** in 80–99% yields, 4:1–>20:1 dr, and 91–99.9% ee (Scheme 18) [93]. The reaction was carried out under mild conditions, and only 1 mol% of *epi*-quininederived squaramide catalyst (**C11**) was used. Later, Yan, Song et al. reported an asymmetric organocatalytic Mannich reaction for the synthesis of *β*-fluoroamine derivatives **65** bearing quaternary C–F centers (Scheme 19) [94]. In the presence of Song’s chiral oligoEG (**C12**) as a cation binding catalyst and KF as a base, *α*-fluoro cyclic ketones **63** were used as nucleophiles to react with *α*-aminosulfones **64** (as the imine surrogates) to give corresponding products in high yields (36–99%) with good enantioselectivities (88–99% ee) and diastereoselectivities (1:2–1:20 *syn:anti*). The proposed reaction mechanism is shown in Scheme 19. Firstly, KF was complexed with catalyst **C12** to form **Int I**, which then complexed with amidinosulfone **64** to generate the **Int II**. Subsequently, the sulfite group on the **64** was removed to obtain the imine-activated **Int III**. The subsequent coordination of potassium enolate (generated in situ from **63** with KF) to the catalyst was followed by its addition to the imine to provide the products **65**. It is noteworthy that the binding of the cation (K^+^) to the catalyst was instrumental in instigating both high reactivity and excellent enantioselectivity during the enantio-determining step by the formation of the chiral cage. Later, a similar catalytic system was applied to the asymmetric Mannich reaction of 3-fluoro-oxindoles **66** with *α*-amidosulfones **64** by the same research group (Scheme 20) [95]. *α*-amidosulfones bearing different aryl and heteroaryl substituents were compatible with generating chiral *α*-fluoro-*β*-amino-oxindole derivatives **67** possessing two contiguous stereogenic centers in 63–97% yields, 1:1–>20:1 dr, and 81–99% ee (*syn*). Unfortunately, alkyl *α*-amidosulfone was unsuitable for this reaction.

**Scheme 21:**
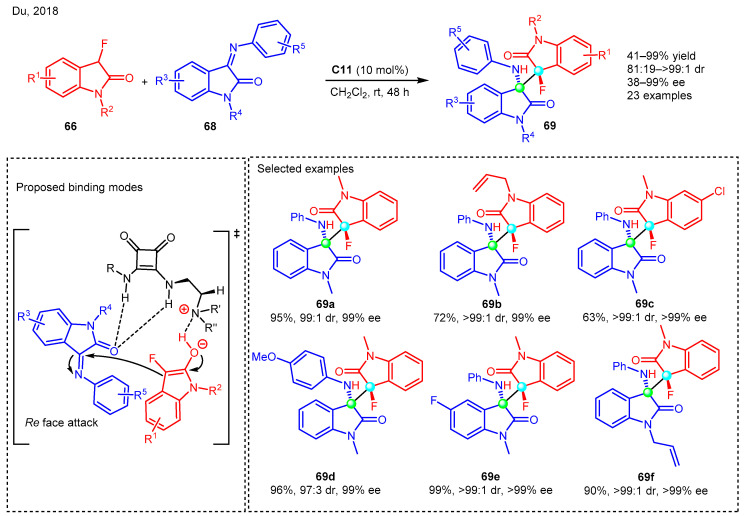
Asymmetric Mannich reaction of 3-fluorooxindoles with isatin-derived imines [96].

In 2018, Du et al. described a bifunctional chiral squaramide (**C11**) catalyzed asymmetric Mannich reactions of 3-fluorooxindoles **66** to isatin-derived imines **68**, which provided fluorinated 3,3’-bisoxindoles **69** bearing two vicinal stereocenters in 41–99% yields, 81:19–>99:1 dr, and 38–99% ee under mild conditions (Scheme 21) [96]. It should be mentioned that the yield, dr, and ee values of the products featuring free N–H were significantly lower. Substituents on N atoms of both substrates were mainly limited to simple methyl substitutions. Additionally, a gram-scale reaction proceeded without the obvious erosion in yield and stereoselectivity.

**Scheme 22:**
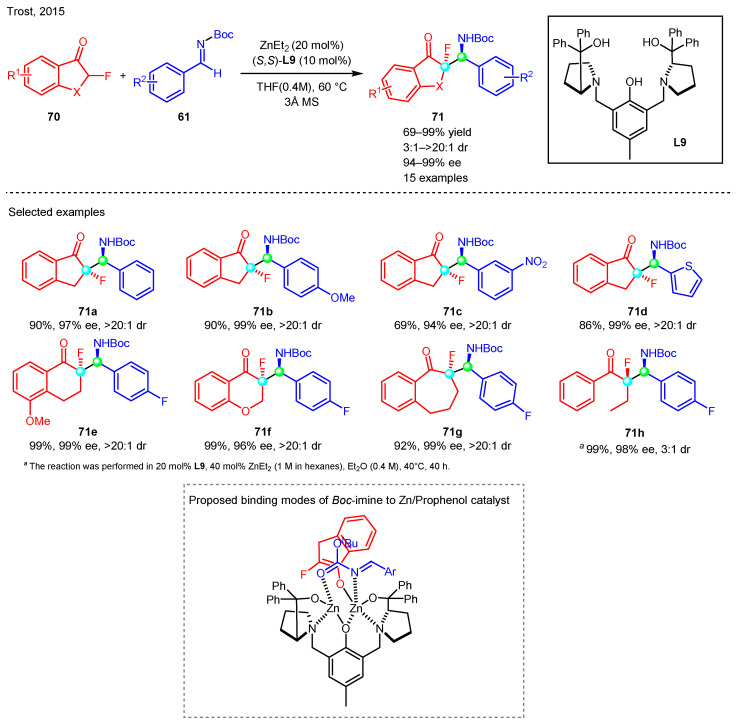
Asymmetric Mannich reactions of fluorinated aromatic ketones with *Boc*-protected aldimines [98].

**Scheme 23:**
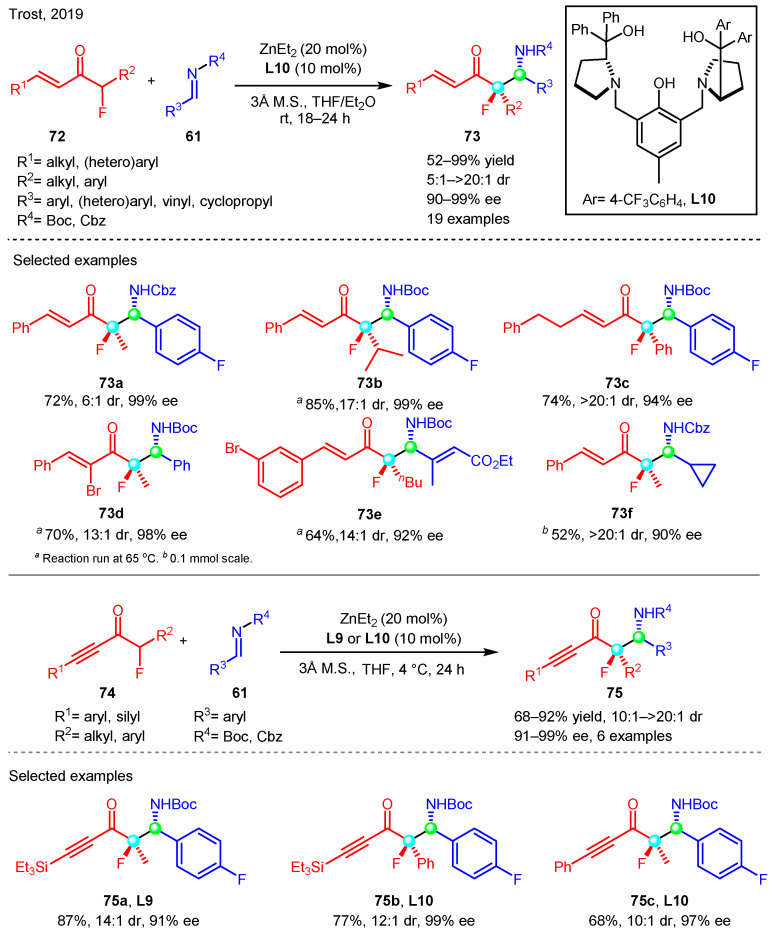
Asymmetric Mannich reactions of branched acyclic vinyl or alkynyl *α*-fluoroketones with aldimines [99].

In 2011, Kim et al. reported a Pd-catalyzed asymmetric Mannich reaction of *α*-fluoro-*β*-ketoesters with *N*-Boc aldimines, which afforded *β*-aminated-*α*-fluoro-*β*-ketoesters bearing two contiguous stereogenic centers with excellent enantioselectivities (up to 99% ee) and good yields (up to 89% yield) [97]. However, the main limitation of this approach was that poor diastereoselectivities were obtained in most cases. In 2015, the Trost group reported a ZnEt_2_/(*R*,*R*)-prophenol (**L9**)-catalyzed highly enantio- and *anti*-diastereoselective Mannich reaction by using α-fluorinated aromatic ketones **70** and *Boc*-protected aldimines **61** as starting materials (Scheme 22) [98]. A series of chiral *β*-fluoroamine **71** were synthesized with 69–99% yields, 94–99% ee, and generally >20:1 dr. In addition, gram-scale studies were also performed at low catalyst loadings (2 mol% **L9**, 4 mol% ZnEt_2_) to obtain the desired product in 98% yield, >20:1 dr, and 99% ee. The proposed binding mode suggests that the *Boc*-protected imines are coordinated between the two zinc centers in a two-point binding manner (Scheme 22). In 2019, the same group realized the asymmetric Mannich reaction between branched acyclic vinyl *α*-fluoro ketones **72** and alkynyl *α*-fluoro ketones **74** and aldimines **61** using similar catalytic systems (Scheme 23) [99]. This was the first example of an asymmetric Mannich reaction involving acyclic α-branched *α*-fluoroketones, affording the corresponding acyclic *β*-fluoro amines featuring two contiguous stereogenic centers in high yields (52–99%), excellent enantioselectivities (90–99% ee), and diastereoselectivities (5:1–>20:1 dr). A variety of vinyl-, cyclopropyl-, aryl-, and heteroaryl-substituted aldimines and various heavily substituted fluoroketones were well worked in this catalytic system.

**Scheme 24:**
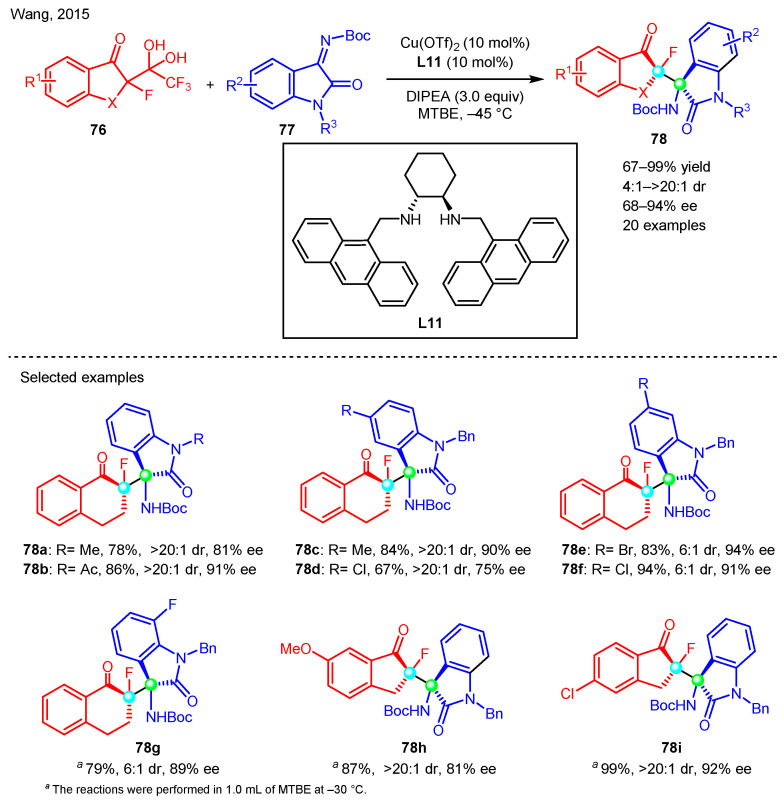
Cu-catalyzed asymmetric detrifluoroacetylative Mannich reaction of 2-fluoro-1,3-diketones/hydrates and isatin-derived ketimines [100].

In 2015, Wang et al. reported a Cu-catalyzed enantioselective detrifluoroacetylative Mannich reaction of 2-fluoro-1,3-diketones/hydrates **76** and isatin-derived ketimines **77** using chiral diamine (**L11**) as ligand (Scheme 24) [100]. Diverse 3-substituted 3-amino-2-oxindoles **78** featuring vicinal tetrasubstituted stereocenters with a quaternary C–F stereogenic carbon were obtained with excellent yields (up to 99%) and good stereochemical results (up to >20:1 dr, 94% ee).

**Scheme 25:**
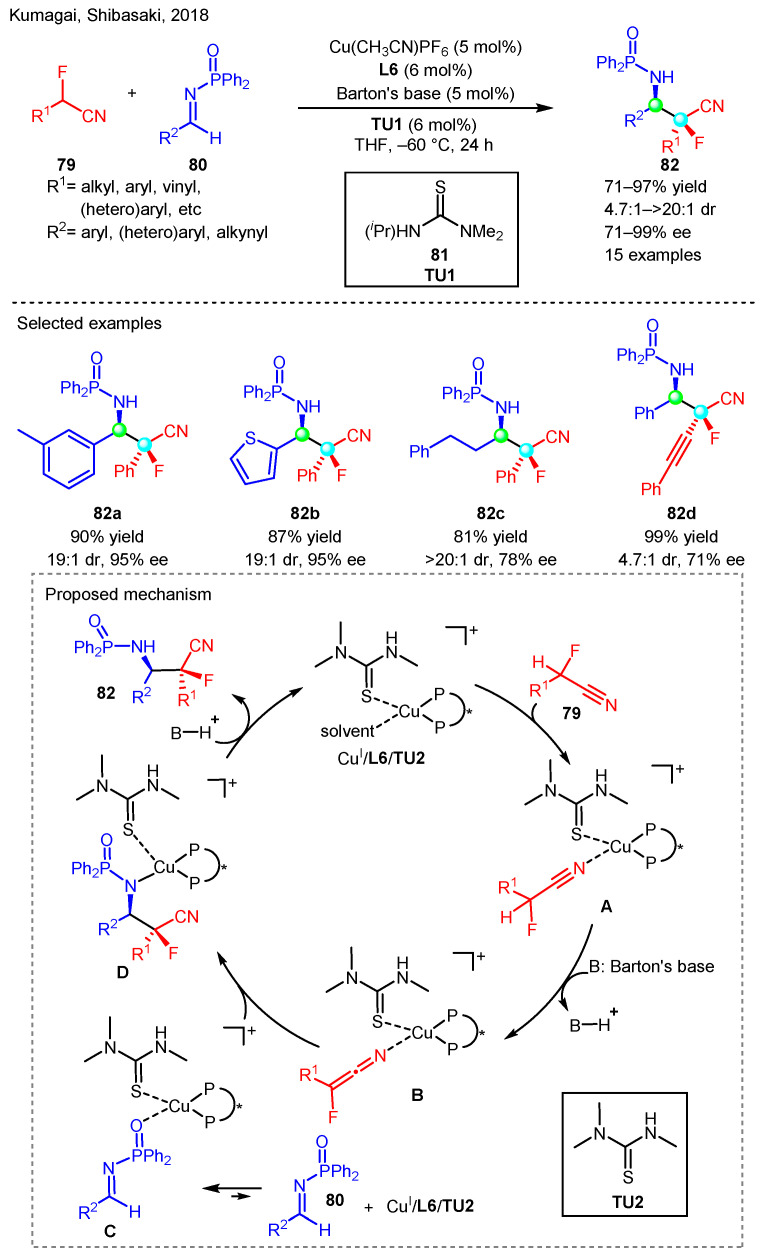
Cu-catalyzed asymmetric Mannich reactions of *α*-fluoronitriles with *N*-diphenylphosphinoyl imines [101].

In 2018, Kumagai and Shibasaki et al. reported a Cu-catalyzed asymmetric Mannich reaction of *α*-fluoronitrile **79** with *N*-diphenylphosphinoyl (*N*-Dpp) imines **80** (Scheme 25) [101]. A series of *β*-amino-*α*-fluoronitriles **82** bearing two adjacent chiral centers were synthesized in 71–97% yields, 4.7:1–>20:1 dr, and 71–99% ee using (*R*)-Segphos (**L6**) as the ligand, achiral thiourea (**TU1**, **81**) as the secondary ligand, and 2-*tert*-butyl-1,1,3,3-tetramethylguanidine (BTMG/Barton’s base) as the base. Further derivatization of the products gave rapid access to *α*-fluorinated-*β*-amino acid derivatives. A gram-scale experiment was carried out without any detrimental effects. Kinetic studies revealed that achiral trisubstituted thioureas enhance the stereoselectivity by binding to the CuI complex. They proposed a plausible reaction mechanism shown in Scheme 25. Firstly, *α*-fluoronitrile coordinates with the preformed CuI/**L6**/**TU2** complex to form complex **A**. Deprotonation of complex **A** promoted by Barton’s base generates CuI- ketenimine complex **B**. The resulting CuI-ketenimine complex **B** reacts with CuI-imine complex **C** to generate CuI amide complex **D** by forming C–C bonds. Finally, CuI amide complex **D** undergoes proton exchange with the protonated Barton’s base to generate the desired product and regenerate the CuI/**L6**/**TU2** complex.

**Scheme 26:**
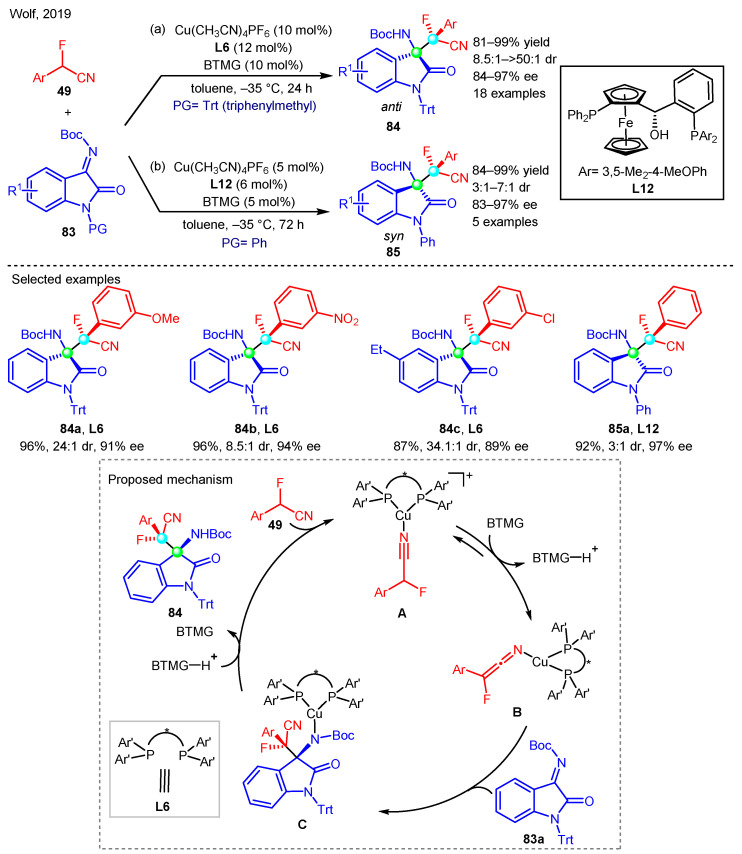
Symmetric Mannich reactions of *α*-fluoro-*α*-arylnitriles with isatin ketimines [102].

Subsequently, Wolf and co-workers used a similar strategy to realize a Cu-catalyzed asymmetric Mannich reaction of α-fluorinated arylacetonitrile **49** with isatin-derived *N*-Boc ketimines **83** (Scheme 26) [102]. *Anti*-diastereomers of multifunctionalized 3-aminooxindoles **84** bearing two adjacent tetrasubstituted stereocenters were obtained in 81–99% yields, 8.5:1–>50:1 dr, and 84–97% ee when **L6** was used as a chiral ligand and the *N*-protecting group of isatin was triphenylmethyl. When **L12** was used as a chiral ligand and the *N*-protecting group of isatin was phenyl, *syn*-diastereomers of 3-aminooxindoles **85** possessing a quaternary carbon–fluorine stereocenter were synthesized in 84–99% yields, 3:1–7:1 dr, and 83–97% ee. The Mannich products can be converted to other useful molecules via selective transformations of oxindole ring opening and nitrile functionality. Additionally, a gram scale experiment was also carried out to probe the utility of this reaction, and the desired *anti*-*α*-fluoro-*β*-aminonitrile was prepared in excellent yield (0.94 g, 99% yield) with 12.7:1 dr and 90% major ee. They proposed a plausible catalytic cycle, shown in Scheme 26. Firstly, *α*-fluorinated arylacetonitrile was coordinated to the (**L6**) Cu(I) complex to form complex **A**, which then underwent reversible deprotonation in the presence of BTMG to form the cuprous keteniminate complex **B**. Subsequently, the isatin ketimine was attacked from the *Si* face of complex **B** to form complex **C** through irreversible C–C bond formation. Finally, complex **C** underwent proton transfer and dissociation to yield the desired product and regenerate the free CuI complex and BTMG.

### 3.4. Michael Addition Reactions

**Scheme 27:**
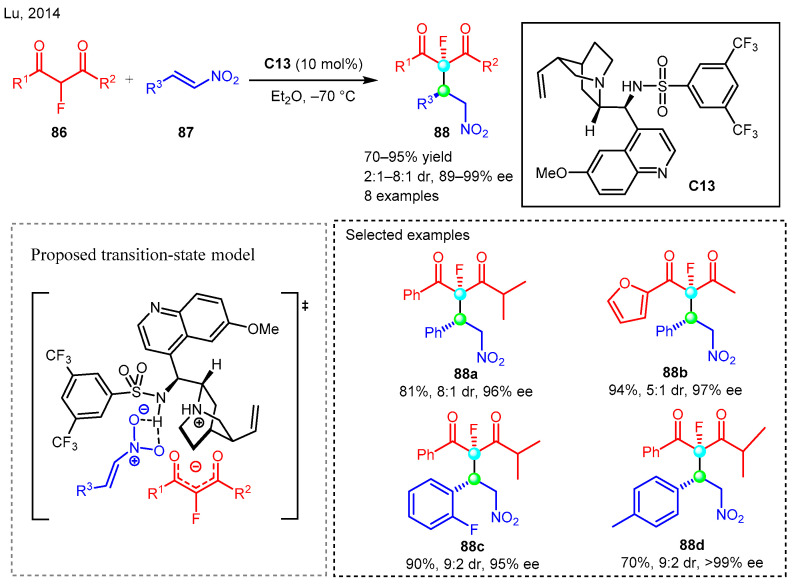
Asymmetric Michael addition reactions of 2-fluoro-1,3 diketones with nitroalkenes [103].

**Scheme 28:**
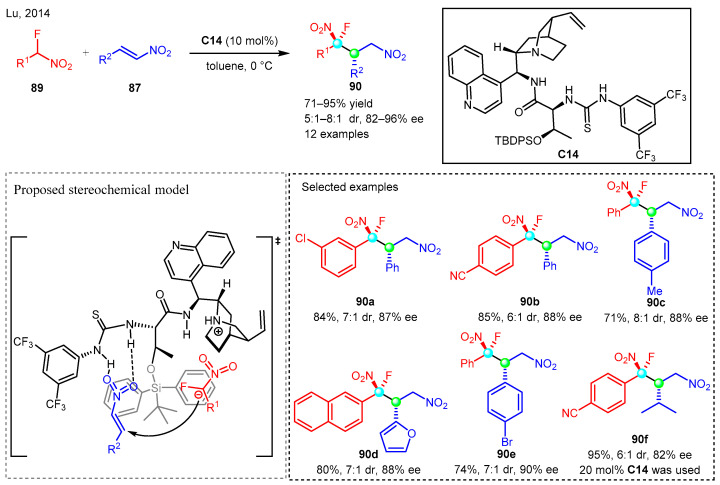
Asymmetric Michael addition reactions of *α*-fluoro-*α*-nitroalkanes with nitroolefins [104].

The asymmetric Michael addition reaction is the other effective method used to construct continuous stereocenters containing fluorine atoms. In 2014, Lu and co-workers reported that quinine-derived sulfonamide **C13** catalyzed asymmetric Michael addition reactions with nitroalkenes **87** as acceptors to react with 2-fluoro-1,3 diketones **86** (Scheme 27) [103]. Several aryl methyl diketones reacted very well with aryl nitroolefins, and the corresponding Michael adducts **88** were obtained in good chemical yields (70–95%), excellent enantioselectivities (89–>99% ee), but moderate diastereoselectivties (2:1–8:1 dr). However, the isopropyl-substituted nitroalkenes substrate showed moderate reactivity and low stereoselectivity with almost no stereoselectivity. The proposed transition-state model is shown in Scheme 27. They believe that the hydrogen-bonding interaction between the nitro group of nitroalkenes and the NH group of sulfonamides is crucial for the observed stereoselectivity. In the same year, an enantioselective Michael addition of *α*-fluoro-*α*-nitroalkanes **89** to nitroolefins **87** was developed by the same group (Scheme 28) [104]. They used amino acid-incorporating multifunctional quinine-derived compound **C14** as the organocatalyst for this reaction, which provided the desired products **90** in 71–95% yields, 5:1–8:1 dr, and 82–96% ee. Different *α*-aryl-*α*-fluoronitromethanes, as well as various aromatic and aliphatic nitroolefins, were suitable substrates for this Michael reaction. However, simple alkyl-substituted *α*-fluoro-*α*-nitroalkanes were unsuitable substrates. Intriguingly, different diastereomers of the Michael addition products could be isolated by a regular flash silica gel chromatographic column. The proposed stereochemical model is shown in Scheme 28. They believed that bifunctional activation of the substrates was important for the observed stereoselectivity.

**Scheme 29:**
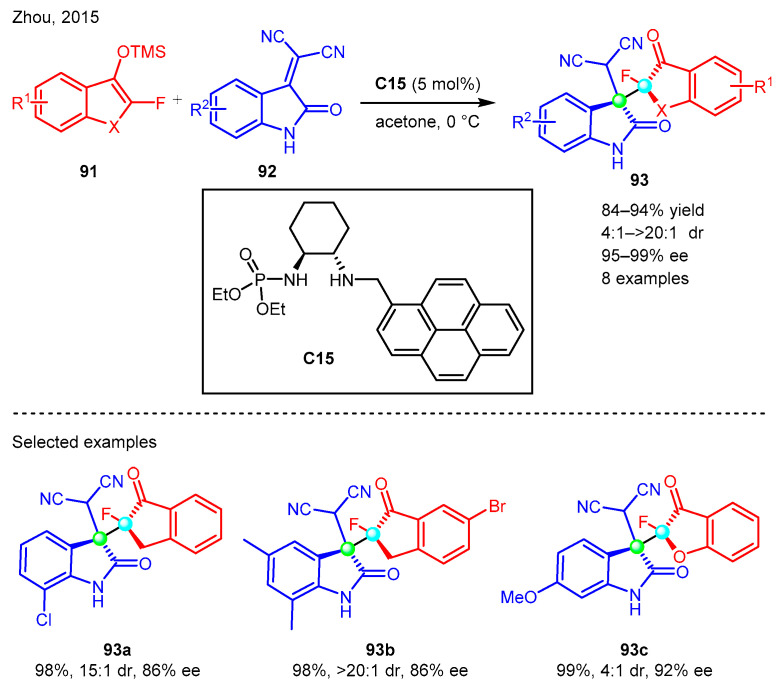
Asymmetric Michael addition reactions of monofluorinated enol silyl ethers with isatylidene malononitriles [105].

In 2015, Zhou et al. developed asymmetric Michael addition of monofluorinated enol silyl ethers **91** to isatylidene malononitriles **92** (Scheme 29) [105]. They used chiral secondary amine phosphoramide **C15** as the organocatalyst for this reaction, which afforded the corresponding monofluorinated oxindole derivatives **93** bearing adjacent and tetrasubstituted carbon stereocenters in excellent chemical yields (95–99%) and high enantioselectivities (84–94% ee) and diastereoselectivities (4:1–>20:1 dr). The product could be transformed into the polycyclic compound in the presence of NaBH_4_ without loss of enantioselectivity.

**Scheme 30:**
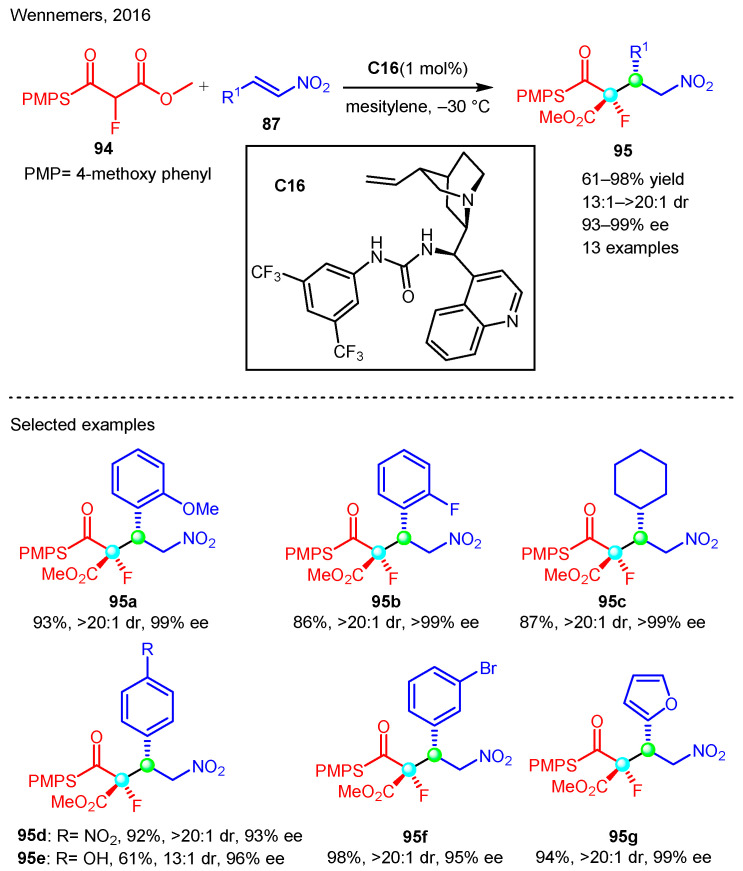
Asymmetric Michael addition reactions of fluorinated monothiomalonates with nitroolefins [106].

**Scheme 31:**
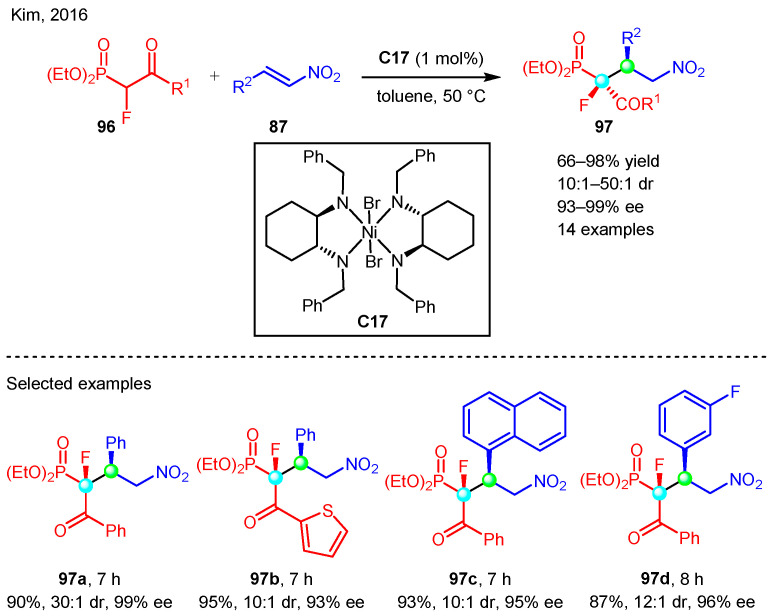
Asymmetric Michael addition reactions of *α*-fluoro-*β*-ketophosphonates with nitroalkenes [107].

In 2016, Wennemers et al. reported an organocatalytic asymmetric Michael addition reaction for the synthesis of *α*-fluoro-*γ*-nitro thioesters **95** bearing adjacent tetrasubstituted and tertiary stereogenic carbon centers (Scheme 30) [106]. They employed fluorinated monothiomalonates (F-MTMs, **94**) as fluoroacetate–enolate equivalents to react with nitroolefins **87** in the presence of 1 mol% of *epi*-cinchonine–urea **C16**. A range of aromatic, heteroaromatic, and even aliphatic nitroolefins were well tolerated and provided the Michael addition products in high chemical yields (61–98%) and excellent stereochemical outcomes (13:1–>20:1 dr, 93–99% ee). Interestingly, the product could be elaborated into fluorinated lactams. In the same year, Kim et al. reported a catalytic asymmetric conjugate addition reaction of *α*-fluoro-*β*-ketophosphonates **96** to nitroalkenes **87** (Scheme 31) [107]. They used 1,2-cyclohexanediamine-coordinated dicationic nickel **C17** as the best chiral catalyst for this reaction, which afforded the corresponding *γ*-nitro *α*-fluorophosphonates derivatives **97** in 66–98% yields, 10:1–50:1 dr, and 93–99% ee. The gram-scale reaction was also conducted to provide the Michael adduct with a high chemical yield (91%) and stereochemical result (12:1 dr, 96% ee).

**Scheme 32:**
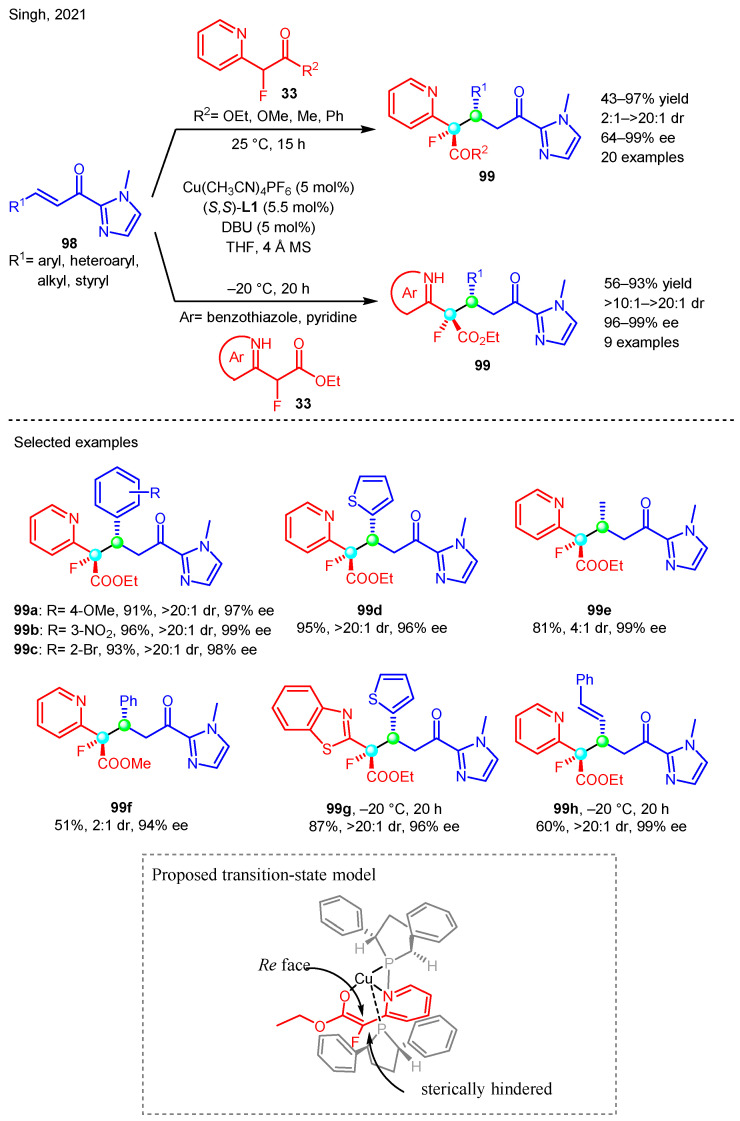
Asymmetric Michael addition reaction of 2-alkylazaarene with unsaturated 2-acylimidazole [108].

Recently, Singh et al. reported a Cu-catalyzed enantioselective Michael addition reaction between alkyl azaarenes **33** and *α*,*β*-unsaturated 2-acyl imidazoles **98** (Scheme 32) [108]. A range of 2-alkyl azaarene derivatives bearing vicinal quaternary–tertiary stereocenters were synthesized with a high level of chemical yields (up to 97%), diastereoselectivities (>20:1), and enantioselectivities (up to 99%) in the presence of 5.5 mol% (*S*,*S*)-**L1** and 5 mol% DBU. The substrates scope was investigated, and *α*,*β*-unsaturated 2-acyl imidazoles with different *ortho*-, *meta*-, and *para*-substitution patterns and heteroaromatic rings substrates were well tolerated, affording excellent yields and enantioselectivities. Surprisingly, under optimized reaction conditions, the yields and selectivity decreased significantly when R^2^ = Me, OMe, Ph. In addition, benzothiazolyl fluoroacetate substrates with electron-donating, electron-withdrawing, halogenated, and heteroaryl substituents proceeded well. 1,6-Michael receptor substrates also reacted smoothly, with the addition reaction occurring only at the 1,4-position. The proposed transition-state model, illustrated in Scheme 32, postulated that the high stereoselectivity was primarily attributed to the binding of chiral Cu(I)-bisphosphines to both the nitrogen of the azaarene ring and the ester carbonyl oxygen of **33**. This binding configuration effectively obstructed one face of the azaarene **33** with the ligand substituent, thereby directing the reaction toward high stereoselectivity.

**Scheme 33:**
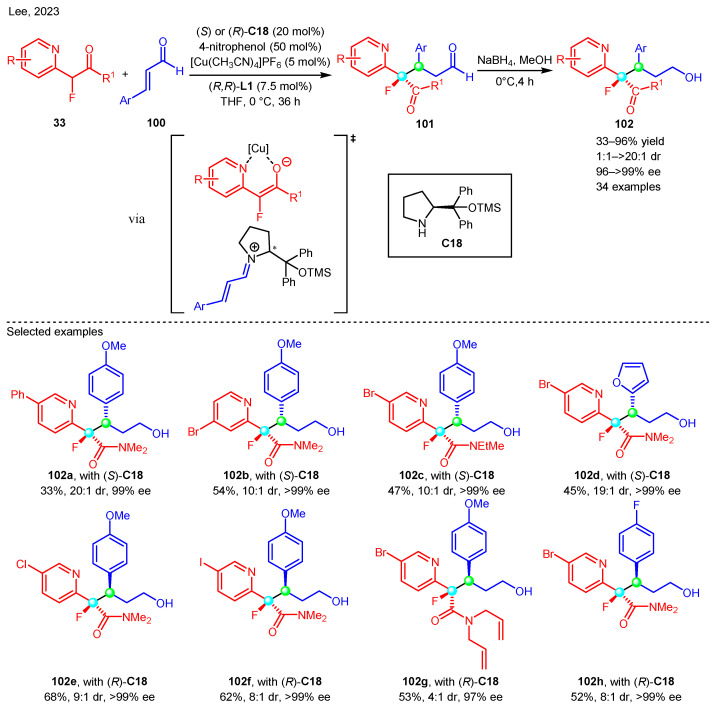
Asymmetric conjugate addition reaction between *α*,*β*-unsaturated aldehydes and *α*-fluoro azaaryl acetamides [109].

In 2023, Lee et al. reported a stereodivergent conjugate addition reaction for the preparation of tertiary alkyl fluorides in adjacent stereogenic pairs (Scheme 33) [109]. They used *α*-fluoro azaaryl acetamides as the *α*-fluoroenolate precursor to react with in situ-generated chiral iminium electrophiles from α,*β*-unsaturated aldehydes **100**. The products, 4-fluorinated 1,5-aldehyde amides **101**, were obtained in generally good yields with high stereocontrol. Most of the adducts were isolated after the reduction of aldehyde to alcohol **102**, during which the diastereomeric ratio did not change. All four stereoisomers of the desired products with vicinal stereocenters were synthesized by variation of the combinations of the enantiomers of phosphine ligand **L1** and amine **C18**. It is worth mentioning that this stereodivergent conjugate addition strategy, combined with aminocatalytic asymmetric *α*-fluorination, enabled the rapid synthesis of all eight stereoisomers of molecules bearing three contiguous stereocenters containing two fluorinated stereocenters.

### 3.5. Aldol Addition Reactions

**Scheme 34:**
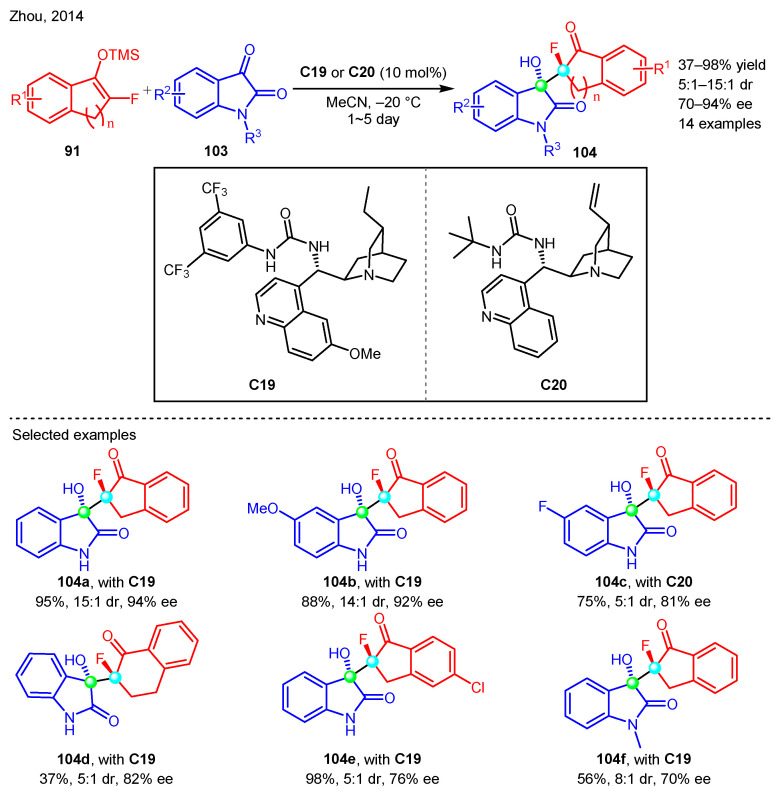
Organocatalytic asymmetric Aldol reaction of monofluorinated silyl enol ethers with isatins [110].

In 2014, Zhou et al. reported an organocatalytic asymmetric aldol addition reaction for the synthesis of optically active 3-hydroxyoxindole derivatives **104** with two adjacent tetrasubstituted carbon stereocenters featuring a C–F bond (Scheme 34) [110]. They used prochiral monofluorinated enol ethers **91** to react with isatins **103** in the presence of cinchona alkaloid-derived bifunctional (thio)urea catalysts **C19** or urea-tertiary amine catalyst **C20**. The solvent had a significant effect on the reaction results, and MeCN was considered the best reaction medium. In the cases of isatins without electron-withdrawing substituents, the reactions were carried out using organocatalyst **C19**, providing the desired products **104** in high chemical yields (37–98% yield), excellent enantioselectivities (70–94% ee), and good diastereoselectivities (5:1–15:1 dr). However, when 5-halo groups substituted isatins were used as substrates, the corresponding products were obtained in 5:1 dr and 81–86% ee in the presence of urea-tertiary amine catalyst **C20**. It should be noted that acyclic monofluorinated silyl enol ether was able to react with isatin under the same conditions, affording the expected product with a moderate yield (46%) and stereochemical outcome (2:1 dr, 81% ee).

**Scheme 35:**
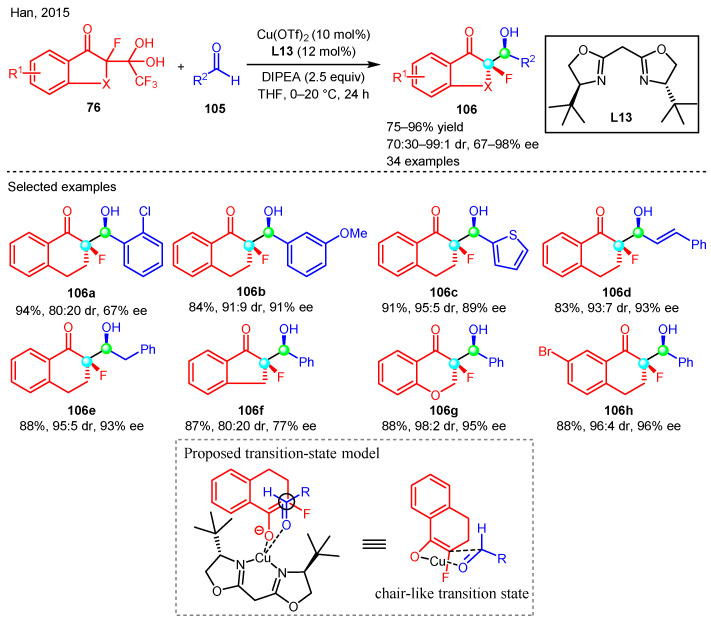
Cu-catalyzed enantioselective detrifluoroacetylative aldol addition reaction of 2-fluoro-1,3-diketones/hydrates with aldehydes [116].

**Scheme 36:**
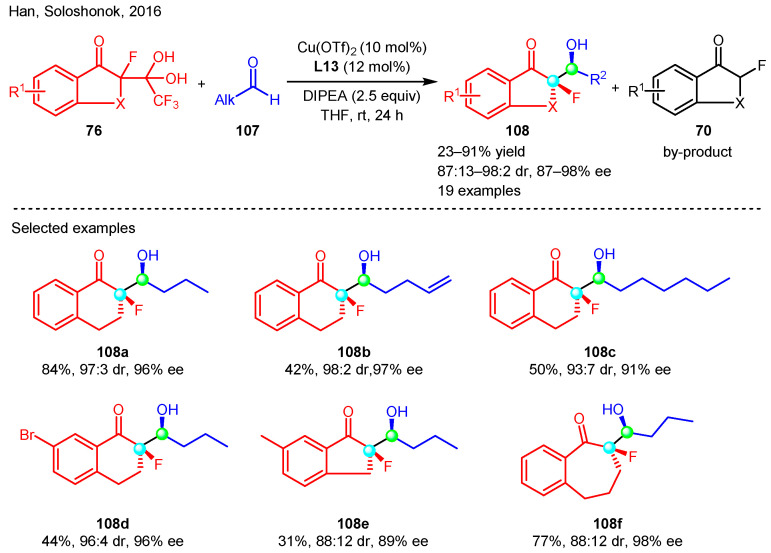
Cu-catalyzed asymmetric detrifluoroacetylative aldol addition reaction of 2-fluoro-1,3-diketones/hydrates with aliphatic aldehydes [117].

**Scheme 37:**
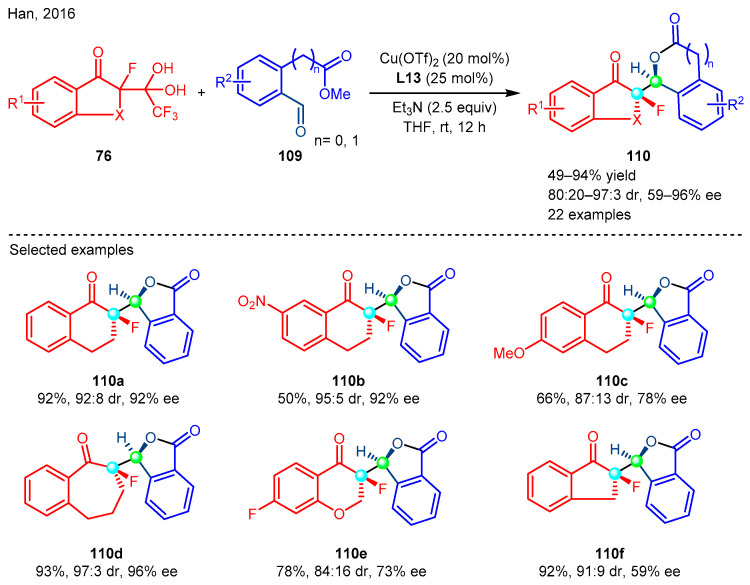
Cu-catalyzed asymmetric cascade aldol-cyclization reaction between detrifluoroacetylatively in situ-generated enolate precursors and ester-aldehydes [118].

**Scheme 38:**
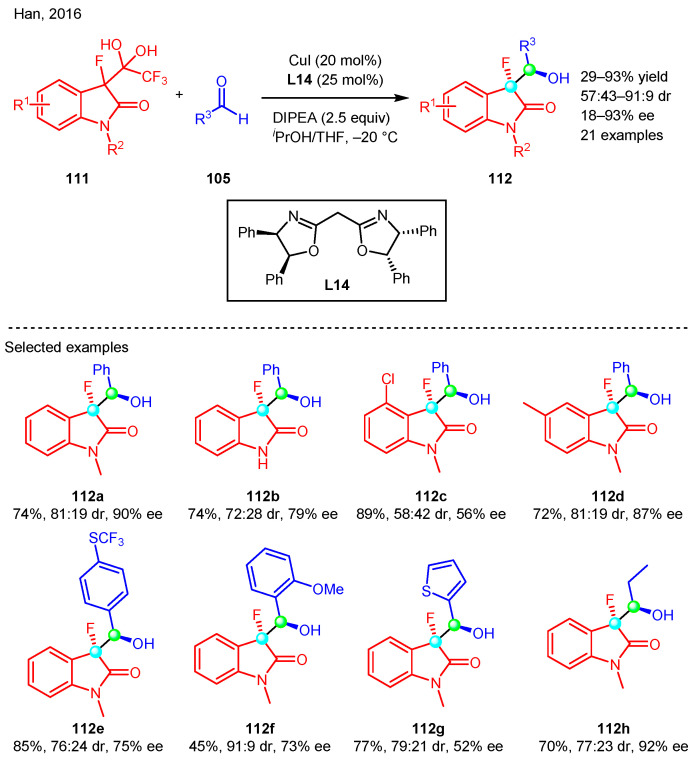
Cu-catalyzed asymmetric aldol reaction between 3-fluoro-2-oxindole derived enolate precursors and aldehydes [119].

In 2011, Colby et al. discovered that 1,1,1,3,3-pentafluoro-2,4-diones could release trifluoroacetic acid to generate *α*,*α*-difluoroenolates via C–C bond cleavage under mild reaction conditions [111,112]. Subsequently, Wolf [113,114], Wu [115], and others [27] used this novel detrifluoroacetylative strategies in an asymmetric aldol addition reaction. In 2015, Han and co-workers developed a Cu-catalyzed enantioselective detrifluoroacetylative aldol addition reaction for the preparation of *α*-fluoro-*β*-hydroxy ketone derivatives **106** with two consecutive stereogenic centers featuring a fluorine atom (Scheme 35) [116]. Under mild conditions, varieties of 2-fluoro-1,3-diketones/hydrates generated from cyclic ketones via two steps could react smoothly with aldehydes in the presence of chiral bidentate ligand **L13** and *N*,*N*-diisopropylethylamine (DIPEA). Various hydrates, arylaldehydes, heteroarylaldehydes, *α*,*β*-unsaturated aldehydes, and phenylacetaldehyde were well tolerated to afford the expected addition products **106** with high yields (75–96% yields) and excellent enantio- and diastereoselectivities (70:30–99:1 dr, 67–98% ee). They proposed a chair-like transition state model (Scheme 35) to explain the observed stereochemical preferences. In 2016, Han, Soloshonok et al. employed a similar strategy to achieve asymmetric detrifluoroacetylative aldol reactions of aliphatic aldehydes **107** (Scheme 36) [117]. Various *α*-fluoro-*β*-hydroxy ketones **108** bearing two contiguous stereogenic centers were obtained with up to 98:2 dr and 98% ee at ambient temperature. They also found that a by-product was generated due to the low reactivity of aliphatic aldehydes. It should be noted that aldehydes containing *β*-branched alkyl groups, such as *^t^*Bu, *^i^*Pr, and *^c^*hexyl, were not suitable substrates. In the same year, Han et al. developed the first example of the Cu-catalyzed enantioselective cascade aldol-cyclization reaction between detrifluoroacetylatively in situ-generated enolate precursors and ester-aldehydes **109** (Scheme 37) [118]. Various bicyclic ketoesters **110** featuring fluorinated quaternary stereogenic were obtained in 49–94% yields, 80:20 dr, and 59–96% ee. However, seven-membered rings containing 1,3-di-keto-hydrate and linear alkyl groups substituted-acyclic 1,3-di-keto-hydrates were ineffective substrates for this catalytic system. Recently, Han, Soloshonok et al. reported a Cu/bisoxazoline ligand **L14-**catalyzed asymmetric detrifluoroacetylative aldol reaction for the synthesis of a range of *α*-fluoro-*β*-hydroxy-indolin-2-ones **112** bearing adjacent tetrasubstituted and tertiary stereogenic centers (Scheme 38) [119]. They used in situ-generated unprotected tertiary enolates derived from 3-(1,1-dihydroxy-2,2,2-trifluoroethyl)-substituted derivatives of 3-fluoro-2-oxindoles **111** as starting materials to react with aldehydes **105**, resulting in the desired products **112** with good yields and satisfactory enantio- and diastereoselectivities. Both benzaldehydes and starting enolate precursors bearing electron-donating and electron-withdrawing groups at almost all positions on the aromatic ring were suitable substrates. Intriguingly, hetero-aromatic, aliphatic aldehydes and enolate precursors containing an unprotected N–H group were well tolerated in this catalytic system.

**Scheme 39:**
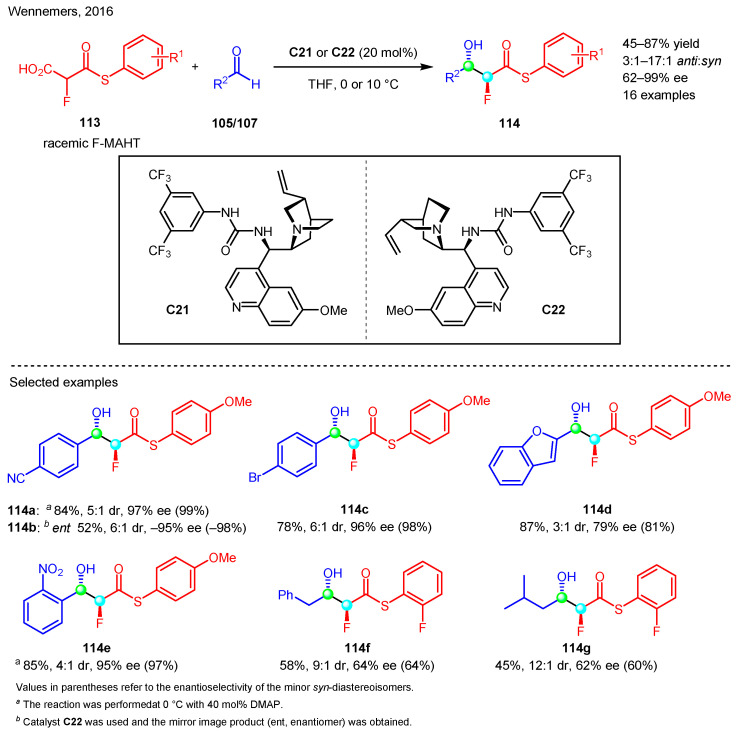
Organocatalytic asymmetric Aldol reaction of fluoromalonic acid halfthioesters with aldehydes [120].

In 2016, Wennemers and Saadi used fluoromalonic acid halfthioesters (F-MAHTs, **113**) as fluoroacetate surrogates for the asymmetric aldol addition reaction (Scheme 39) [120]. The quinidine–urea catalyst **C21** was chosen as the best organocatalyst for this aldol reaction, which could catalyze this reaction smoothly to give the corresponding fluorinated thioesters **114** with up to 87% yields, 17:1 dr, and 99% ee. Both the more-reactive aromatic aldehydes and the less-reactive aliphatic aldehydes were well tolerated to react with electron-rich 4-methoxythiophenol-derived F-MAHT or 2-fluorothiophenol-derived F-MAHT. They also utilized the *pseudo*-enantiomer quinine-derived catalyst **C22** for this reaction and provided the mirror image product with high yields and stereoselectivities, similar to the corresponding enantiomers. Gladly, the *anti*- and *syn*-diastereoisomers could be easily separated by column chromatography, and the minor *syn*-isomers were obtained in high enantioselectivities equal to the corresponding major *anti*-isomers.

**Scheme 40:**
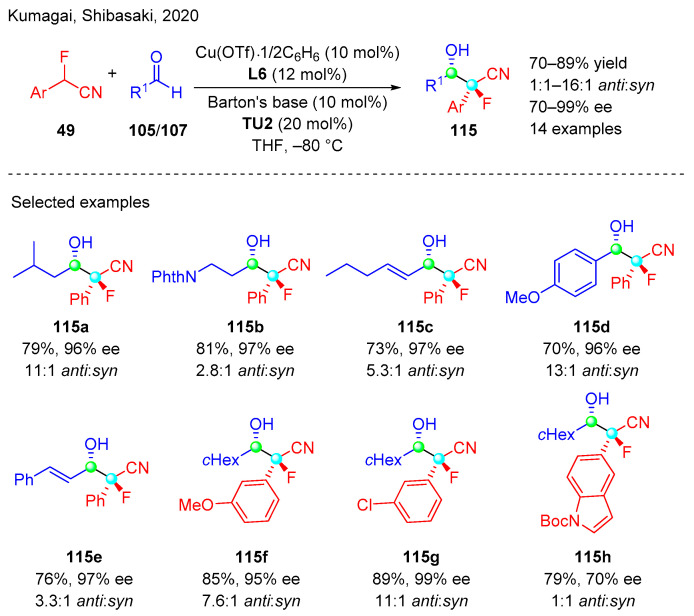
Cu-catalyzed asymmetric Aldol reaction of *α*-fluoronitriles with aldehydes [121].

In 2020, Kumagai, Shibasaki et al. developed a Cu-catalyzed asymmetric aldol addition reaction between *α*-fluoronitriles **49** and aldehydes **105**/**107** (Scheme 40) [121]. The reaction used **L6** as the chiral ligand and conducted the reaction at −80 °C in the presence of asymmetrical achiral thioureas **TU2** as a secondary ligand and Barton’s base. Nitriles with electron-donating and electron-withdrawing substituents and aliphatic and aromatic aldehydes were well tolerated to give the desired *α*-fluoro-*β*-hydroxynitriles **115** featuring two vicinal stereogenic centers with 70–89% yields, 1:1–16:1 dr, and 70–99% ee. The stereoselectivity of this chiral CuI/Barton’s base catalytic system could be significantly enhanced by the combined use of trimethylthiourea **TU2**.

### 3.6. Miscellaneous Reactions

**Scheme 41:**
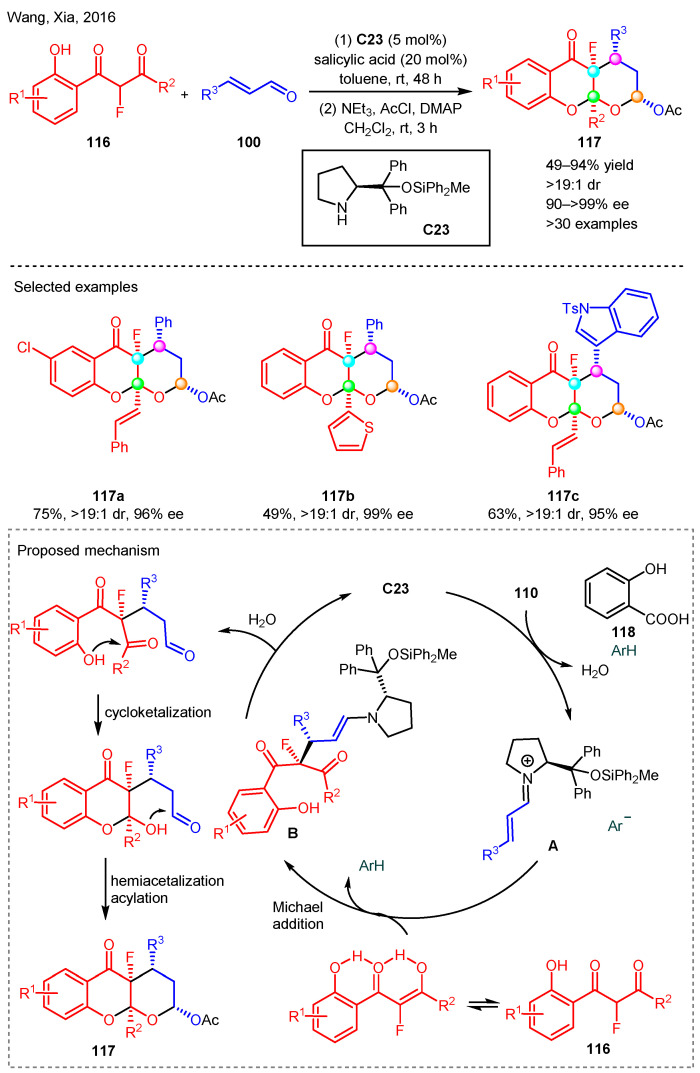
Synthesis of fluorinated tricyclic chromanones via asymmetric tandem reaction [122].

In 2016, Wang, Xia et al. reported an organocatalytic asymmetric tandem Michael addition/cycloketalization/hemiacetalization and acylation transformations for the preparation of optically pure fluorinated *O*,*O*-acetal fused tricyclic derivatives with multiple adjacent stereogenic centers using *α*,*β*-unsaturated aldehydes **100** and 2-fluoro-1-(2-hydroxyaryl)-1,3-dione **116** as starting materials (Scheme 41) [122]. The chiral amine **C23** was chosen as the best catalyst, which efficiently catalyzed the reaction to give the desired products **117** with excellent stereochemical outcome (>19:1 dr, 90–>99% ee). A plausible mechanism for this asymmetric tandem reaction is shown in Scheme 41. Firstly, with the assistance of salicylic acid **118**, the chiral amine **C23** and cinnamaldehyde generated the chiral iminium ion **A**. Then, 2-fluoro-1-(2-hydroxyaryl)-1,3-dione **116** attacked the *Si* face of chiral iminium **A** and underwent a Michael addition reaction to generate the intermediate **B**. After the hydrolysis of intermediate **B**, the catalyst **C23** was recovered. Subsequently, the intermediate **B** underwent cyclic ketonization/hemiacetalization and acylation reactions to give the target products **117**.

**Scheme 42:**
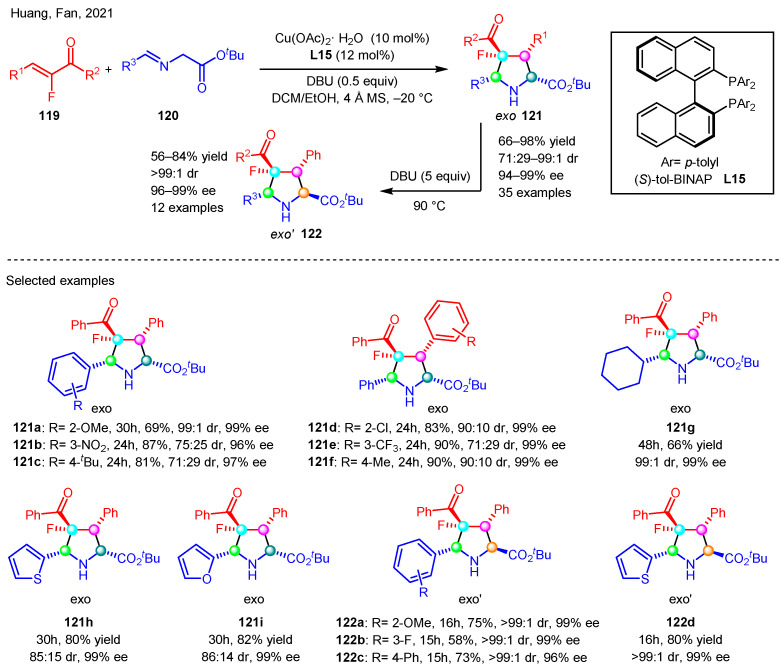
Cu-catalyzed asymmetric 1,3-dipolar cycloaddition reactions [123].

In 2021, Huang, Fan et al. reported a Cu^II^-catalyzed enantioselective 1,3-dipole cycloaddition of glycine imines **120** with *α*-fluoro-*β*-aryl-*α*,*β*-unsaturated arylketones **119** (Scheme 42) [123]. Diverse chiral *exo*-pyrrolidine derivatives **121** with four contiguous stereogenic centers containing a fluorinated quaternary stereogenic center at the C4 position could be prepared in high yields (up to 98%) with excellent enantio- and diastereoselectivity (up to 99:1 dr and 99% ee) in the presence of a Cu(OAc)_2_·H_2_O/(*S*)-*tol*-BINAP **L15** catalyst, DBU, and 4 Å molecular sieves in DCM/EtOH at −20 °C. These *exo*-products could be further converted to the unusual *exo’* adducts **122** in good yields (56–84%) with excellent stereochemical outcomes (>99:1 dr, 96–99% ee) at 5.0 equivalents of DBU and 90 °C.

**Scheme 43:**
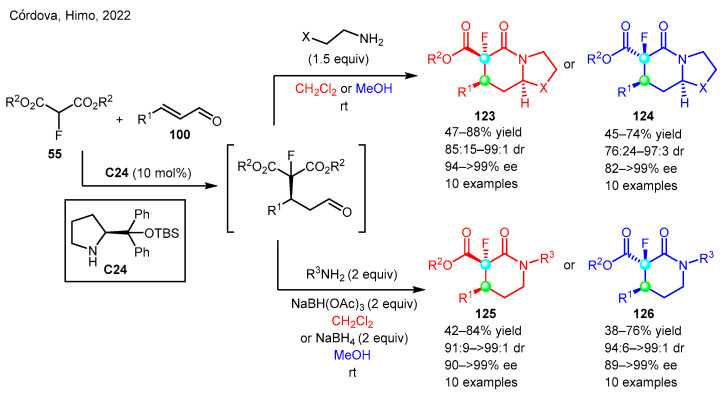
Stereoselective synthesis of *α*-fluoro-*δ*-lactams [124].

In 2022, Córdova and Himo et al. reported a solvent dependency in the stereoselective intramolecular amidation reaction of chiral 5-inofunctionalized-2-fluoromalonate ester derivatives in the presence of a chiral catalyst **C24** (Scheme 43) [124]. In solvents with dielectric constants (*ε*) less than 10 (e.g., CH_2_Cl_2_), *δ*-lactams **123**/**125** with a *syn*-configuration between adjacent tertiary carbon and fluorine-containing quaternary stereocenters were generally generated. In solvents with *ε* > 15 (e.g., MeOH), the corresponding *δ*-lactams **124**/**126** with *anti*-configuration were formed. This catalytic asymmetric cascade reaction combined with solvent-directed stereoselective reactions provided a novel strategy for the stereodivergent synthesis of all possible stereoisomers of heterocyclic compounds with two vicinal stereogenic centers featuring C–F quaternary stereocenters.

**Scheme 44:**
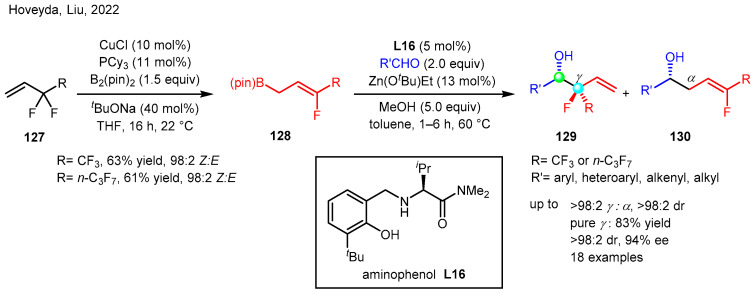
Organozinc complex-catalyzed asymmetric synthesis of homoallylic alcohols [125].

In 2022, Hoveyda, Liu et al. developed an in situ-generated Zn(O*^t^*Bu)Et/aminophenol (**L16**) complex catalyzed highly regio-, diastereo-, and enantioselective reactions between fluoro-substituted allylboronates **128** and aldehydes (Scheme 44) [125]. Diverse aryl-, heteroaryl-, alkenyl-, and alkyl-substituted aldehydes were well tolerated in this catalytic system to give homoallylic alcohols **129** with adjacent stereogenic carbon centers containing a trifluoromethyl- and a fluoro-substituted stereogenic center. It is worth noting that the electrophilicity of aldehydes affected *γ* selectivity, and the more electron-rich aldehydes provided higher *γ* selectivity. In contrast, *γ*:*α* selectivity was lower for the more electron-deficient aldehydes.

**Scheme 45:**
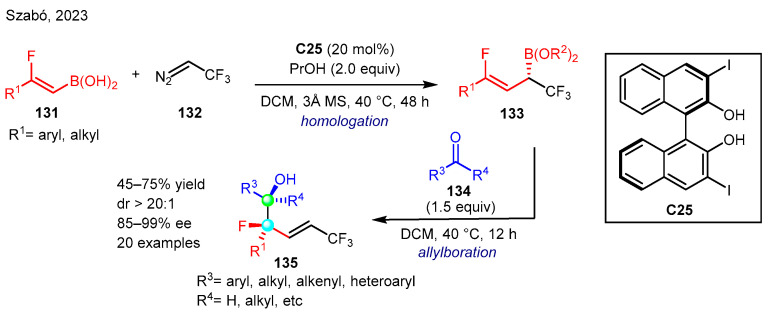
Catalytic homologation/allylboration sequence for the synthesis of *β*-fluorohydrin [126].

In 2023, Szabó et al. developed a catalytic asymmetric one-pot homologation-allylboration sequence for the preparation of *β*-fluorohydrins **135** with adjacent C–F and C–O stereocenters in 45–75% yields, >20:1 dr, and 85–99% ee (Scheme 45) [126]. The target products were obtained via the homologation of trisubstituted fluoroalkenes **131** by using (*R*)-iodo-BINOL **C25** as a catalyst, followed by the allylboration of ketone, aldehyde, and imine.

**Scheme 46:**
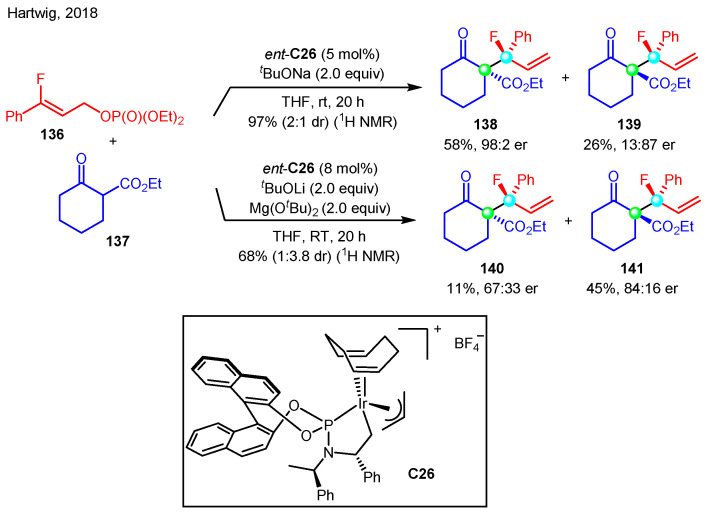
Iridium-catalyzed diastereo- and enantioselective fluoroalkylation of ethyl 2-oxocyclohexanecarboxylate [127].

**Scheme 47:**
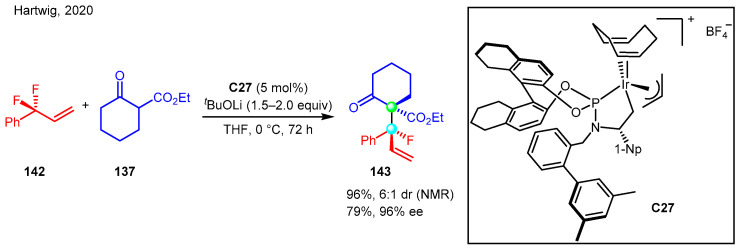
Iridium-catalyzed defluorinative alkylation reaction of 1,1-difluoroallylbenzene [128].

We also noted that Hartwig et al. reported an iridium-catalyzed enantioselective allylic substitution reaction between 3-fluoro allylic electrophiles and soft carbon nucleophiles. Encouragingly, diastereoselective fluoroalkylation of ethyl 2-oxocyclohexanecarboxylate **137** with 3-fluoroallyl phosphate **136** in the presence of the *ent*-**C26** catalyst processed smoothly to give desired diastereomers with two consecutive stereogenic centers in suitable yields with moderate diastereo- and enantioselectivity (Scheme 46) [127]. In 2020, Hartwig et al. developed the iridium-catalyzed desymmetrization of allylic difluoromethylene groups to afford optically pure tertiary allylic fluorides **143** with high yield, regioselectivity, and enantioselectivity via the activation of a single C–F bond. The participation of a prochiral *β*-keto ester in the reaction gave allyl fluorides with two contiguous, fully substituted stereocenters containing one fluorine atom with good diastereoselectivity (6:1 dr) and excellent enantioselectivity (96% ee) (Scheme 47) [128].

## 4. Conclusions

Fluorinated compounds are important molecules that are widely used in material sciences, pharmaceuticals, agrochemicals, and other fields. Particularly, fluorine-containing compounds with complex and variable structures and multiple continuous stereocenters have attracted much attention. This review summarizes the important research progress in the synthesis of alkyl fluorinated compounds bearing multiple contiguous stereogenic centers in recent years, including the asymmetric electrophilic fluorination and asymmetric elaboration of fluorinated substrates (such as allylic alkylation reactions, hydrofunctionalization reactions, Mannich addition reactions, Michael addition reactions, Aldol addition reactions, and miscellaneous reactions), and with an emphasis on synthetic methodologies, substrate scopes, and reaction mechanisms. The success of these methods is attributed to the ingenious design of catalysts/substrates/reaction modes. We hope that this review will provide new ideas for the discovery of more common and efficient new catalysts/methods/reagents for the synthesis of high-value fluorinated compounds and their derivatives.

## Data Availability

Not applicable.

## Abbreviations

Entry	Abbreviation	Full name of compound
1	FDA	Food and Drug Administration.
2	PET	Positron emission tomography.
3	CADA	Catalytic asymmetric dearomatization.
4	Selectfluor	1-chloromethyl-4-fluoro-1,4-diazoniabicyclo[2.2.2]octane bis(tetrafluoroborate).
5	NFSI	*N*-Fluorobenzenesulfonimide.
6	BINOL	2,2’-Dihydroxy-1,1’-binaphthyl.
7	PS	Proton sponge.
8	Boc	*t*-Butyloxy carbonyl.
9	Cbz	Benzyloxycarbonyl.
10	Fmoc	9-Fluorenylmethyloxycarbonyl.
11	TIP	Thiazoline iminopyridine.
12	pyr·9HF	Pyridine·9HF.
13	*m*-CPBA	*meta*-chloroperbenzoic acid.
14	(*R*,*R*)-BPE	(-)-1,2-Bis((2*R*,5*R*)-2,5-diphenylphospholano)ethane.
15	DBU	1,8-Diazabicyclo[5.4.0]undec-7-ene.
16	Me-THQphos	(2*R*)-1-(11b*R*)-Dinaphtho[2,1-d:1’,2’-f][1–3]dioxaphosph epin-4-yl-1,2,3,4-tetrahydro-2-methylquinoline.
17	TBD	1,5,7-Triazabicyclo[4.4.0]dec-5-ene.
18	THF	Tetrahydrofuran.
19	2,5-DPBQ	2,5-diphenylquinone.
20	(*S*)-*t*-Bu-PHOX	(*S*)-4-(*tert*-Butyl)-2-(2’-(diphenylphosphanyl)-[1,1’-biphenyl]-2-yl)-4,5-dihydrooxazole.
21	OligoEG	Oligo ethylene glycol.
22	*N*-Dpp	*N*-diphenylphosphinoyl.
23	**TU1**	3-Isopropyl-1,1-dimethylthiourea.
24	BTMG	2-*tert*-Butyl-1,1,3,3-tetramethylguanidine.
25	**TU** **2**	1,1,3-Trimethylthiourea.
26	F-MTMs	Fluorinated monothiomalonates.
27	DIPEA	*N*,*N*-Diisopropylethylamine.
28	*^t^*Bu	*tert*-Butyl.
29	*^i^*Pr	Isopropyl.
30	*^c^*hexyl	Cyclohexyl.
31	F-MAHTs	Fluoromalonic acid halfthioesters.
32	(*S*)-*tol*-BINAP	(*S*)-(–)-2,2’-Bis(di-p-tolylphosphino)-1,1’-binaphthyl.
33	DCM	Dichloromethane.
34	EtOH	Ethanol.
35	MeOH	Methanol.
36	*ε*	Dielectric constants.
37	(*R*)-iodo-BINOL	(*R*)-3,3’-Diiodo-[1,1’-binaphthalene]-2,2’-diol.
38	TBS	*tert*-Butyldimethylsilyl.
39	Bs	Benzenesulfonyl.
40	TIPS	Triisopropylsilyl.
41	LiHMDS	Lithium bis(trimethylsilyl)amide.
42	MTBE	Methyl *tert*-butyl ether.
43	TMS	Trimethylsilyl.
44	DMAP	4-Dimethylaminopyridine.
45	TBDPS	*t*-Butyl-diphenylsilyl.
